# Curcumin activates Nrf2 through PKCδ-mediated p62 phosphorylation at Ser351

**DOI:** 10.1038/s41598-021-87225-8

**Published:** 2021-04-19

**Authors:** Jee-Yun Park, Hee-Young Sohn, Young Ho Koh, Chulman Jo

**Affiliations:** Division of Brain Disease Research, Department for Chronic Disease Convergence Research, Korea National Institute of Health, 187 Osongsaengmyeong2-ro, Osong-eup, Cheongju-si, Chungcheongbuk-do 363-951 South Korea

**Keywords:** Biochemistry, Cell biology, Molecular biology

## Abstract

Curcumin, a phytochemical extracted from *Curcuma longa* rhizomes, is known to be protective in neurons via activation of Nrf2, a master regulator of endogenous defense against oxidative stress in cells. However, the exact mechanism by which curcumin activates Nrf2 remains controversial. Here, we observed that curcumin induced the expression of genes downstream of Nrf2 such as HO-1, NQO1*,* and *GST-mu*1 in neuronal cells, and increased the level of Nrf2 protein. Notably, the level of p62 phosphorylation at S351 (S349 in human) was significantly increased in cells treated with curcumin. Additionally, curcumin-induced Nrf2 activation was abrogated in *p62* knockout (−/−) MEFs, indicating that p62 phosphorylation at S351 played a crucial role in curcumin-induced Nrf2 activation. Among the kinases involved in p62 phosphorylation at S351, PKCδ was activated in curcumin-treated cells. The phosphorylation of p62 at S351 was enhanced by transfection of PKCδ expression plasmid; in contrast, it was inhibited in cells treated with *PKCδ-*specific siRNA. Together, these results suggest that PKCδ is mainly involved in curcumin-induced p62 phosphorylation and Nrf2 activation. Accordingly, we demonstrate for the first time that curcumin activates Nrf2 through PKCδ-mediated p62 phosphorylation at S351.

## Introduction

Curcumin, a major component extracted from the rhizome of *Curcuma longa*, has shown a broad range of pharmacological activities including antioxidant, anti-inflammatory, antibacterial, and antitumor activities^1–3^. Curcumin has been confirmed to cross the blood–brain barrier^4,5^, and has shown neuroprotective activities in models of neurological disorders such as stroke and traumatic brain injury^6–9^. Curcumin also appears to have protective activity in neurodegenerative disease models, including Alzheimer’s disease (AD) and Parkinson’s disease (PD) models^1,10,11^. Studies in the last decade have shown that curcumin can impact various molecules and induce several cell signaling pathways such as AKT, Nrf2 (nuclear factor erythroid-2 related factor 2), NF-*κ*B, p38, Jak/STAT, and AMPK pathways^2,12^. Among the signaling pathways activated by curcumin, accumulating evidence demonstrates that curcumin-activated Nrf2 plays a crucial role in neuroprotection by counteracting oxidative stress and brain edema^6–8,13^. However, the exact mechanism by which Nrf2 is activated by exogenous curcumin remains to be explored.

Nrf2 is normally present in the cytoplasm of mammalian cells, where it is consistently ubiquitinylated and targeted for degradation by the ubiquitin proteasome system. Nrf2 degradation is mainly initiated by the association with the redox-sensitive Kelch-like ECH-associated protein 1 (Keap1), an adaptor protein of Cul3/Rbx1 E3 ubiquitin ligase complex. In response to a variety of oxidative and electrophilic insults, the interaction of Keap1 with Nrf2 is weakened by chemical modification, which protects Nrf2 from degradation. Nrf2 then accumulates in the nucleus where it heterodimerizes with one of the small musculoaponeurotic fibrosarcoma (MAF) proteins, which bind to antioxidant response element (ARE) sequences in genes targeted by Nrf2, thus increasing their transcription^14,15^. The target genes of Nrf2 have been recently found to be about 250 in humans, and include those that not only encode antioxidant and detoxification enzymes, but also regulate inflammation and proteostasis^16^.

p62/sequestosome (SQSTM) 1 (hereafter referred to as p62) is a stress-inducible scaffold protein involved in diverse cellular processes such as selective autophagy, apoptosis, and adipogenesis^17,18^. Notably, p62 can interact with Keap1 through the KIR (Keap1-interacting region) domain which includes the sequence (349-DPSTGE-354)^19,20^. Under basal conditions, the binding-dissociation constant of p62 with Keap1 is lower than that of Nrf2. Several stresses can induce the phosphorylation of p62 at S351 (S349 in humans), which markedly increases its binding affinity to Keap1^21^. Consequently, phosphorylated p62 disrupts the interaction between Keap1 and Nrf2, which is required for Nrf2 ubiquitination and its subsequent degradation by the proteasome, thus stabilizing and activating Nrf2^19–21^. The mechanism of activating Nrf2 by the direct interaction of p62 with Keap1 is non-canonical, in contrast to the previously described activation of Nrf2 by Keap1 modification^19,22^. To date, multiple kinases, including mTORC1, Tak1 (TGF-β activated kinase 1), and PKCδ/VPS34, have been shown to participate in the phosphorylation of p62 at S351^21,23,24^. Intriguingly, p62 expression is induced by Nrf2, due to the presence of ARE sequences in the p62 promoter, thus creating a positive feedback loop^25^.

Here, we examined whether curcumin could activate Nrf2 in neuronal cells. We report for the first time that curcumin activates Nrf2 through PKCδ-mediated p62 phosphorylation at S351. Our results provide a crucial scientific clue towards understanding of the biological activity of curcumin in neuronal cells.

## Results

### Curcumin activates Nrf2

**Figure 1:**
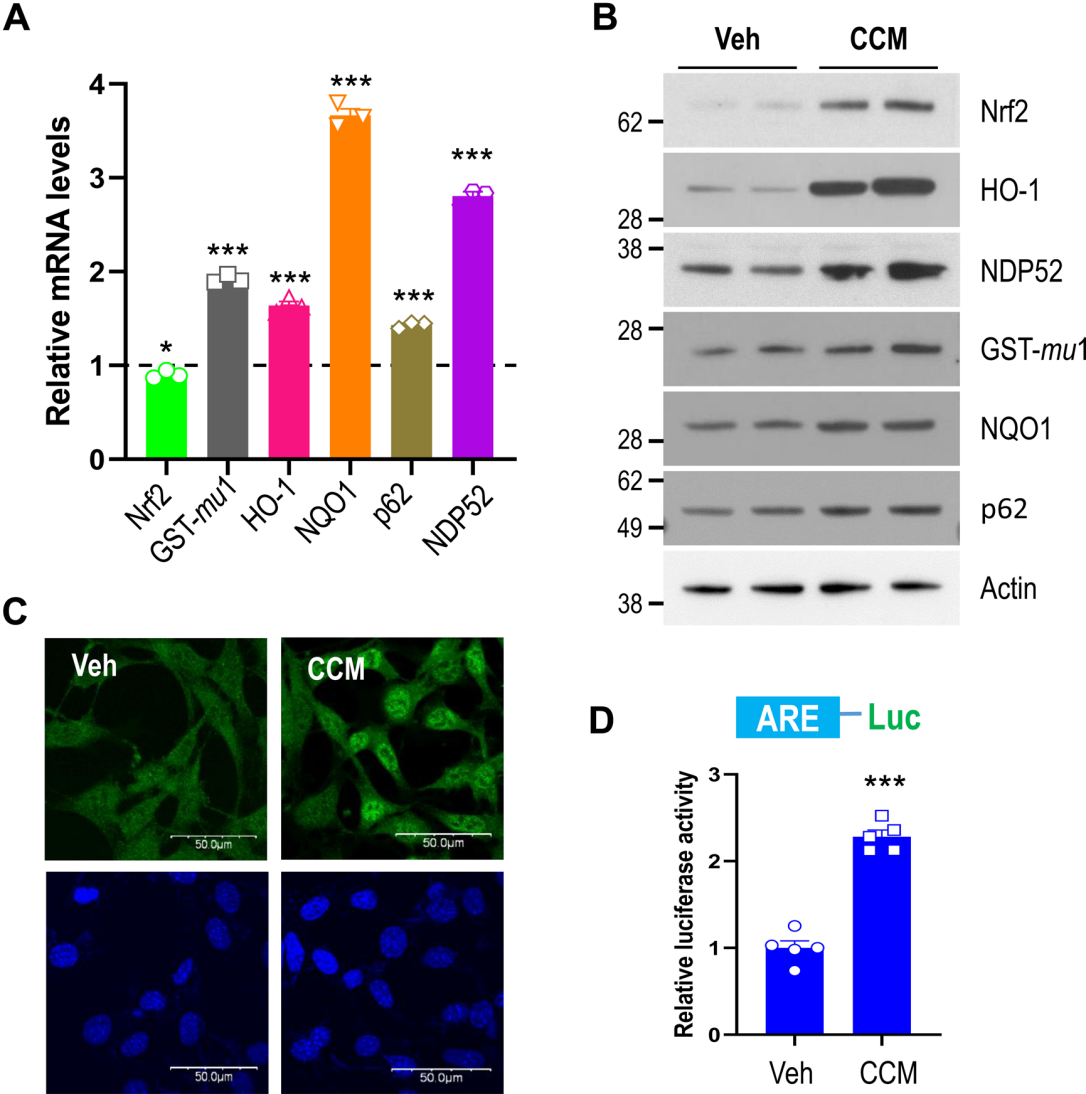
Curcumin activates Nrf2 signaling. (**A**) Mouse cortical cells were treated with either DMSO or 10 μM curcumin for 12 h. The mRNA levels of Nrf2-response genes were analyzed by qRT-PCR as described in the Methods. The bar graph shows the relative mRNA level of genes in the curcumin-treated group compared to the DMSO group. (**B**) The protein levels of Nrf2-response genes in the cells were analyzed by immunoblotting using anti-Nrf2, HO-1, NDP52, GST-*mu*1, NQO1, p62, and actin antibodies, respectively. Full blots are provided in Supplementary Fig. S11. (**C**) Mouse cortical cells were treated with either DMSO (Veh) or 10 μM curcumin (CCM) for 6 h. The cells were fixed with 4% paraformaldehyde and immunostained using anti-Nrf2 antibody. Fluorescence signals were observed using a confocal laser scanning microscope. (**D**) Mouse cortical cells were transiently transfected with the ARE-Luc reporter and TK-Renilla plasmids. After treatment with either DMSO (Veh) or 10 μM curcumin (CCM) for 12 h, the cells were assayed for the luciferase activity. Data shown are mean ± S.E. of three independent experiments and were analyzed using the Student’s *t*-test. (*p < 0.05, ***p < 0.001).

To examine whether curcumin activates Nrf2 in neuronal cells, cells were treated with curcumin for 12 h. Treatment of neuronal cells with 10 μM curcumin did not alter their viability (Supplementary Fig. 1). As shown in Fig. 1A, the mRNA levels of genes downstream of Nrf2 such as GST-*mu*1, HO-1, NQO1, p62, and NDP52 were significantly increased in cells treated with curcumin, compared to untreated controls. The expression levels of these proteins were also increased (Fig. 1B). Additionally, Nrf2 protein levels increased highly in the presence of curcumin (Fig. 1B). Immunohistochemical staining using a Nrf2-specific antibody revealed that Nrf2 was mainly localized in the nuclei of cells treated with curcumin (Fig. 1C). Nrf2 levels also increased in nuclear fractions of cells treated with curcumin, compared to control cells not treated (Supplementary Fig. 2). The transcriptional activity of the promoter containing the triple ARE sequences increased 2.3-fold in cells treated with curcumin (Fig. 1D). Together, these results suggest that Nrf2 is activated in neuronal cells by curcumin.

### Nrf2 activation is dependent on p62

**Figure 2:**
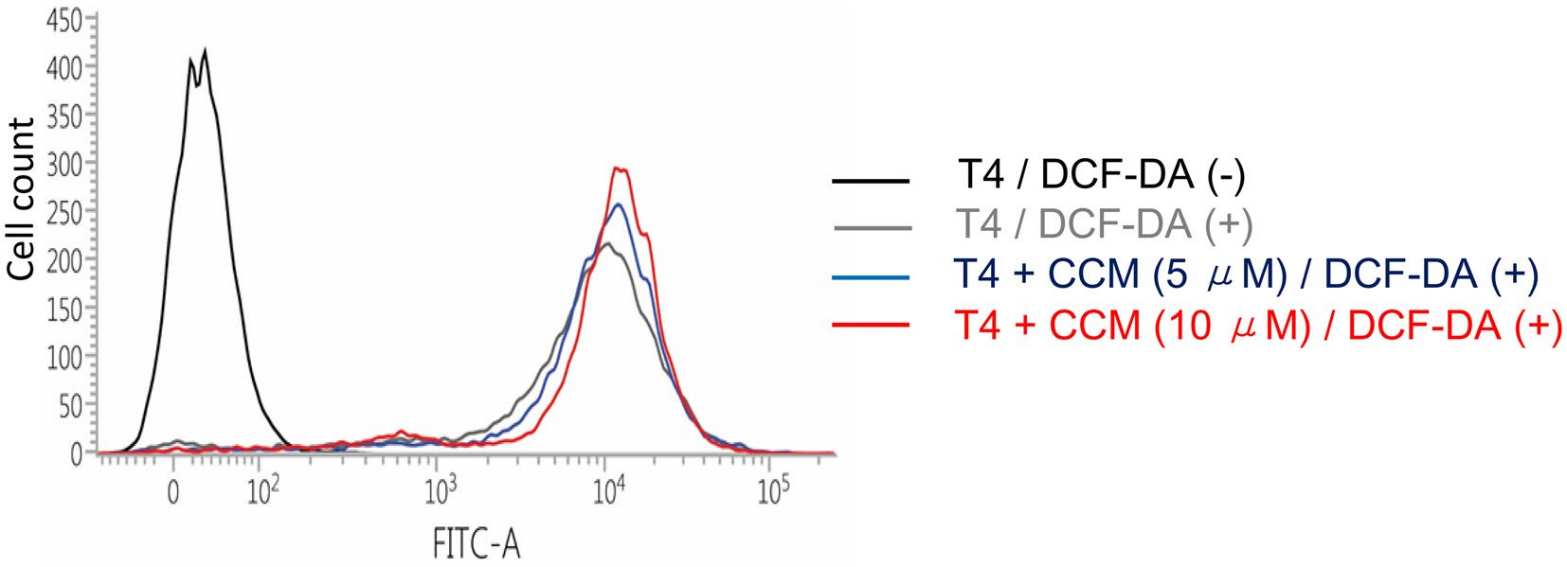
Curcumin does not increase endogenous ROS. Mouse cortical cells were treated with DMSO, 5 μM or 10 μM curcumin (CCM) for 6 h followed by treatment with DMSO (DCF-DA (−)) or 10 μM DCF-DA (DCF-DA (+)) in DMEM without serum for 30 min. The fluorescence signal was detected at the FITC channel on a FACS Instrument.

**Figure 3:**
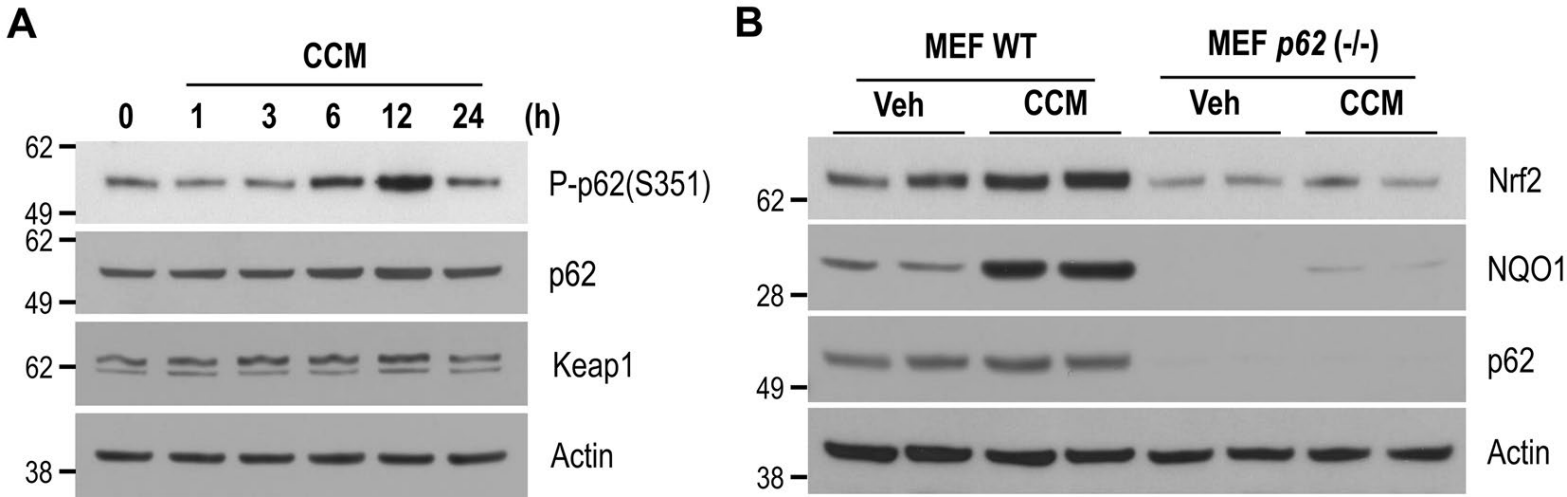
Curcumin-mediated Nrf2 activation is dependent on p62. (**A**) Mouse cortical cells were treated with DMSO (0 h) or 10 μM curcumin (CCM) for the indicated times. The levels of phosphorylated p62 (S351), p62, Keap1, and actin proteins were analyzed by immunoblotting using anti-phospho p62 (S349), p62, Keap1, and actin antibodies, respectively. (**B**) MEFs were treated with DMSO (Veh) or 10 μM curcumin (CCM) for 12 h. The protein levels of Nrf2, NQO1, p62, and actin were analyzed by immunoblotting using anti-Nrf2, NQO1, p62, and actin antibodies, respectively. Full blots are provided in Supplementary Fig. S11.

A few studies have suggested that curcumin induces autophagic cell death or apoptosis by inducing endogenous reactive oxygen species (ROS) production^26,27^. We sought to confirm whether increased endogenous ROS in curcumin-treated neuronal cells elicited Nrf2 activation. To do this, we analyzed the cellular fluorescence intensity of DCF-DA, a cell-permeable indicator for ROS, following the treatment of neuronal cells with curcumin. There was no significant difference between cells treated with curcumin and untreated control cells (Fig. 2), thereby indicating that curcumin itself did not induce cellular ROS production in neuronal cells. As shown in Fig. 1A,B, curcumin significantly increased both the mRNA and protein levels of p62, and increased Nrf2 protein levels. As p62 phosphorylation at S351 can help stabilize and activate Nrf2 protein^21^, we examined the level of phosphorylated p62 at S351 using a S351 phospho-specific p62 antibody. Results showed that the level of p62 protein phosphorylated at S351 was dramatically increased after 6 h or 12 h of curcumin treatment (Fig. 3A). Therefore, we postulated that increased phosphorylation of p62 at S351 by curcumin could stabilize and activate Nrf2 in neuronal cells.

To determine the extent to which p62 was involved in curcumin-mediated Nrf2 activation, we examined the effect of curcumin treatment on the level of Nrf2 protein in *p62* knockout (−/−) MEFs. As shown in Fig. 3B, the previously observed increase in Nrf2 protein level was abated in curcumin-treated *p62* (−/−) MEFs. Moreover, the NQO1 protein was not significantly induced in the *p62* (−/−) MEFs, in contrast to the wild-type MEFs.

**Figure 4:**
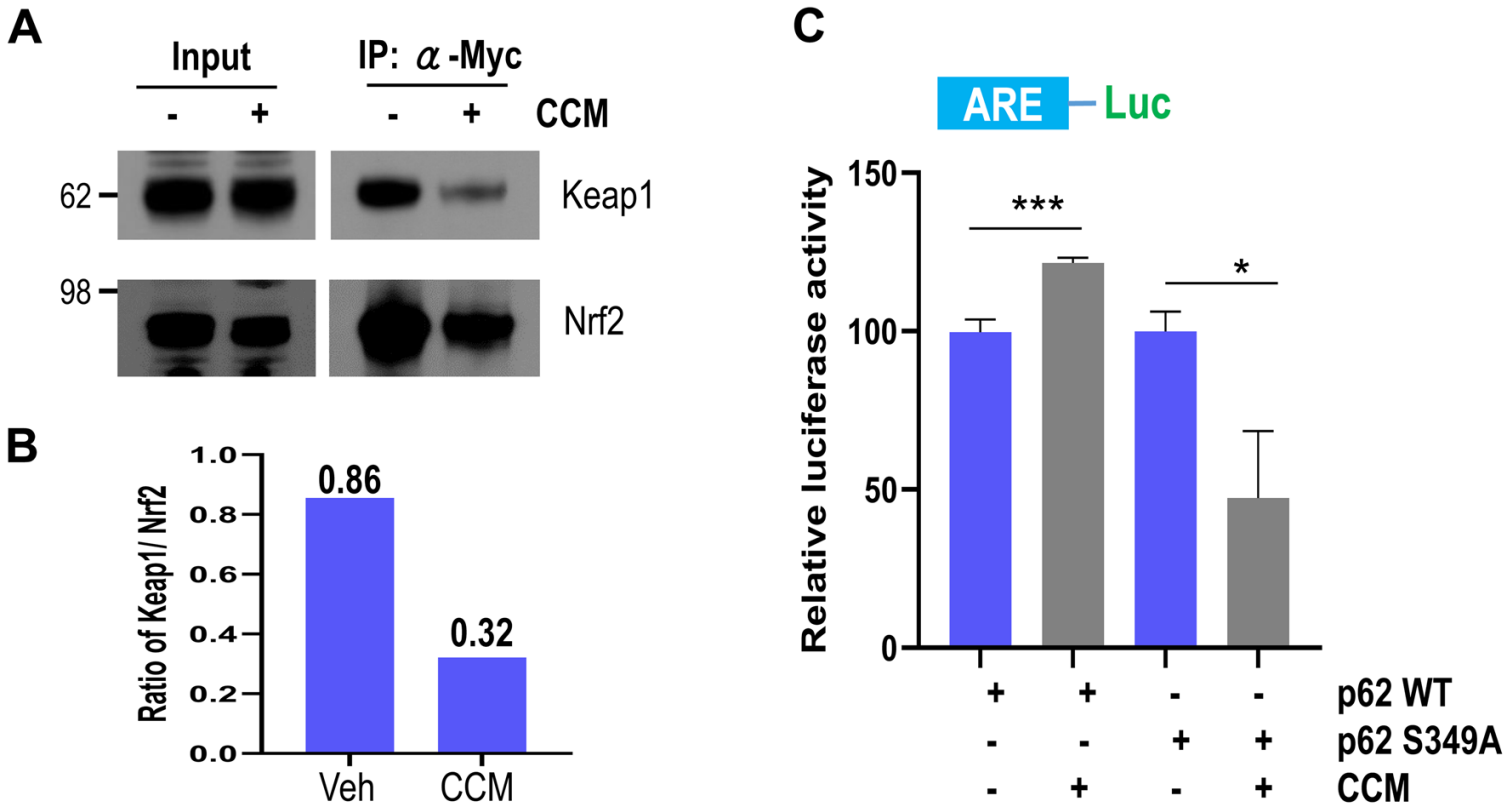
Curcumin-mediated Nrf2 activation is dependent on the phosphorylation of p62 on S351. (**A**) HEK293 cells were transiently transfected with the Myc-Nrf2 expression plasmid, and then treated with DMSO (−) or 10 μM curcumin (+) for 12 h. The cell lysates were used for Nrf2 immunoprecipitation using an anti-Myc antibody. The protein level of Keap1 co-immunoprecipitated with Nrf2 was examined by immunoblotting using an anti-Keap1 antibody. Full blots are provided in Supplementary Fig. S11. (**B**) The bar graph shows the relative ratio of Keap1 against Nrf2 co-immunoprecipitated with the Myc antibody in cells treated with curcumin (CCM) or not. (**C**) HEK293 cells were transiently co-transfected with the ARE-Luc reporter and TK-Renilla plasmids along with the Myc-p62 wild-type or mutant (S349A) plasmid. After treatment with either DMSO (−) or 10 μM curcumin (+) for 12 h, the cells were assayed for the luciferase activity. Data shown are mean ± S.E. of three independent experiments and were analyzed using the Student’s *t*-test. (*p < 0.05, ***p < 0.001).

To further examine whether curcumin-induced increase of p62 phosphorylation at S351 could affect the association between Keap1 and Nrf2, we investigated their interaction using the co-immunoprecipitation test. As shown in Fig. 4A,B, the protein amount of Keap1 co-immunoprecipitated with Nrf2 in the cells treated with curcumin was about 38% compared to that in control cells not treated. Also, curcumin-induced increase in the promoter activity containing ARE elements was ameliorated in the presence of p62 mutant (S349A) (Fig. 4C). Thus, our results strongly suggest that curcumin-mediated Nrf2 activation is highly dependent on p62 and its phosphorylation at S351.

### PKCδ phosphorylates p62 at S351

p62 has been reported to be phosphorylated at S351 by several kinases such as mTORC1, Tak1, CK1, and VPS34-dependent PKCδ under several conditions^17^. To determine which kinase was involved in curcumin-induced p62 phosphorylation at S351, we examined the levels of p62 phosphorylation at S351 following pretreatment with several kinase-specific inhibitors, including Torin1 (for mTORC1), PP242 (for mTORC1), (5Z)-7-Oxozeanol (for Tak1), CKI7 (for CK1), and SAR405 (for VPS34). As shown in Supplementary Fig. 3, there was no significant difference in the level of p62 phosphorylation at S351 in cells pretreated with Tak1, CK1, VPS34, and other inhibitors. However, mTORC1 inhibitors were the exception, suggesting that mTORC1 might be involved in curcumin-mediated augmentation of p62 phosphorylation at S351. Therefore, we investigated whether mTORC1 was activated in neuronal cells in the presence of curcumin, by measuring the phosphorylation levels of mTORC1 and ULK1, a protein downstream of mTORC1. Surprisingly, the levels of mTORC1 phosphorylation at S2448 and ULK1 phosphorylation at S757 were largely unchanged by curcumin (Supplementary Fig. 4), thereby indicating that mTORC1 might not be activated by curcumin in neuronal cells.

**Figure 5:**
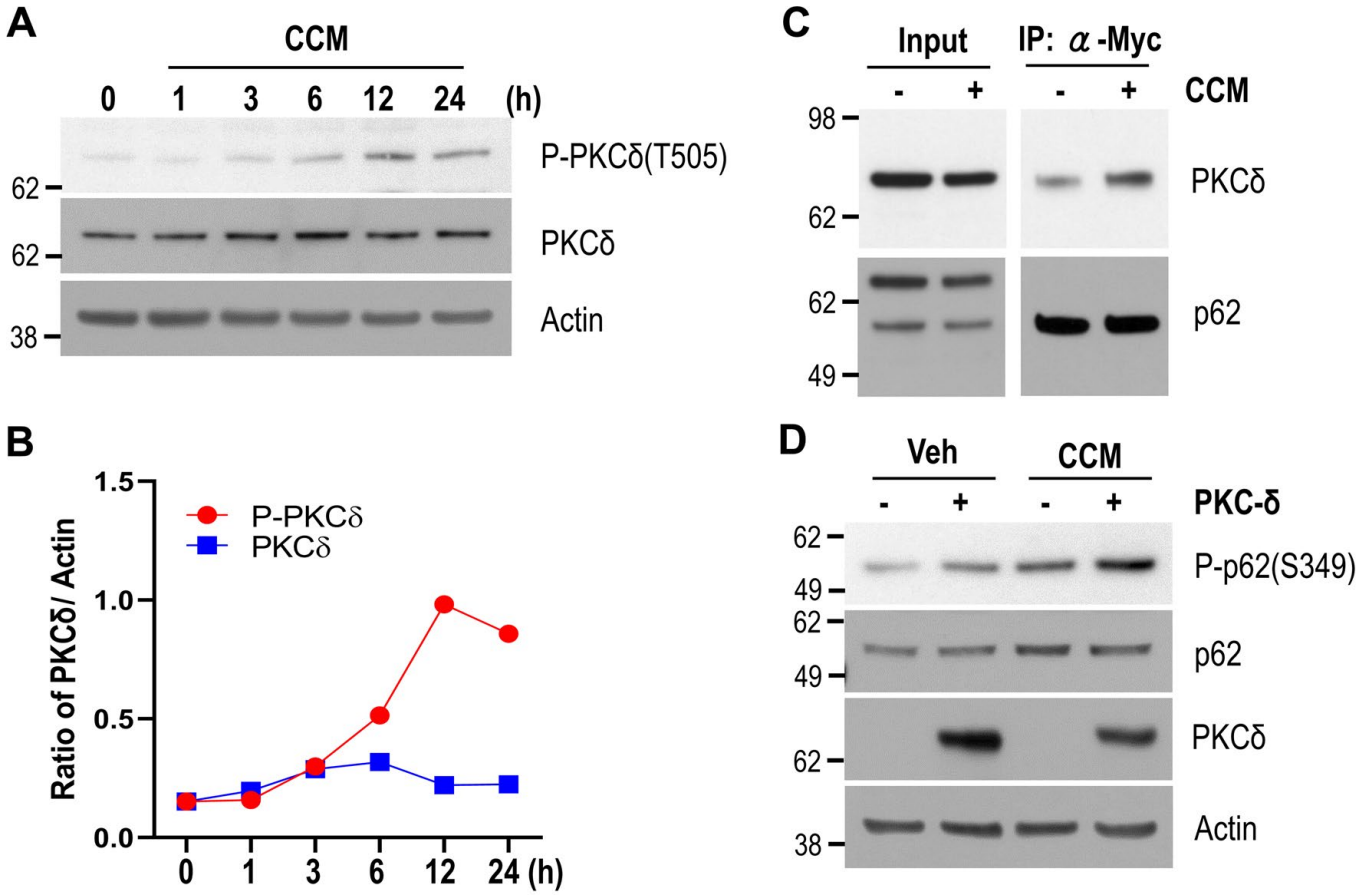
Curcumin activates PKCδ. (**A**) Mouse cortical cells were treated with DMSO (0 h) or 10 μM curcumin (CCM) for the indicated times. The levels of PKCδ phosphorylation at T505 was analyzed by immunoblotting using an anti-phospho PKCδ (T505) antibody. The protein levels of PKCδ and actin were analyzed by immunoblotting using anti-PKCδ, and actin antibodies, respectively. (**B**) The line graph shows the relative ratio of phosphorylated PKCδ at T505 or PKCδ normalized to that of actin. (**C**) HEK293 cells were transiently transfected with Myc-p62 and HA-PKCδ expression plasmids, and then treated with DMSO (−) or 10 μM curcumin (+) for 6 h. The cell lysates were used for p62 immunoprecipitation using an anti-Myc antibody. The protein level of PKCδ co-immunoprecipitated with p62 was examined by immunoblotting using an anti-HA antibody. (**D**) HEK293 cells were transiently transfected with the HA-PKCδ expression plasmid and treated with DMSO (Veh) or 10 μM curcumin (CCM) for 12 h. The levels of phosphorylated p62 (S349), p62, PKCδ, and actin proteins were examined by immunoblotting using anti-phospho p62 (S349), p62, HA, and actin antibodies, respectively. Full blots are provided in Supplementary Fig. S11.

**Figure 6:**
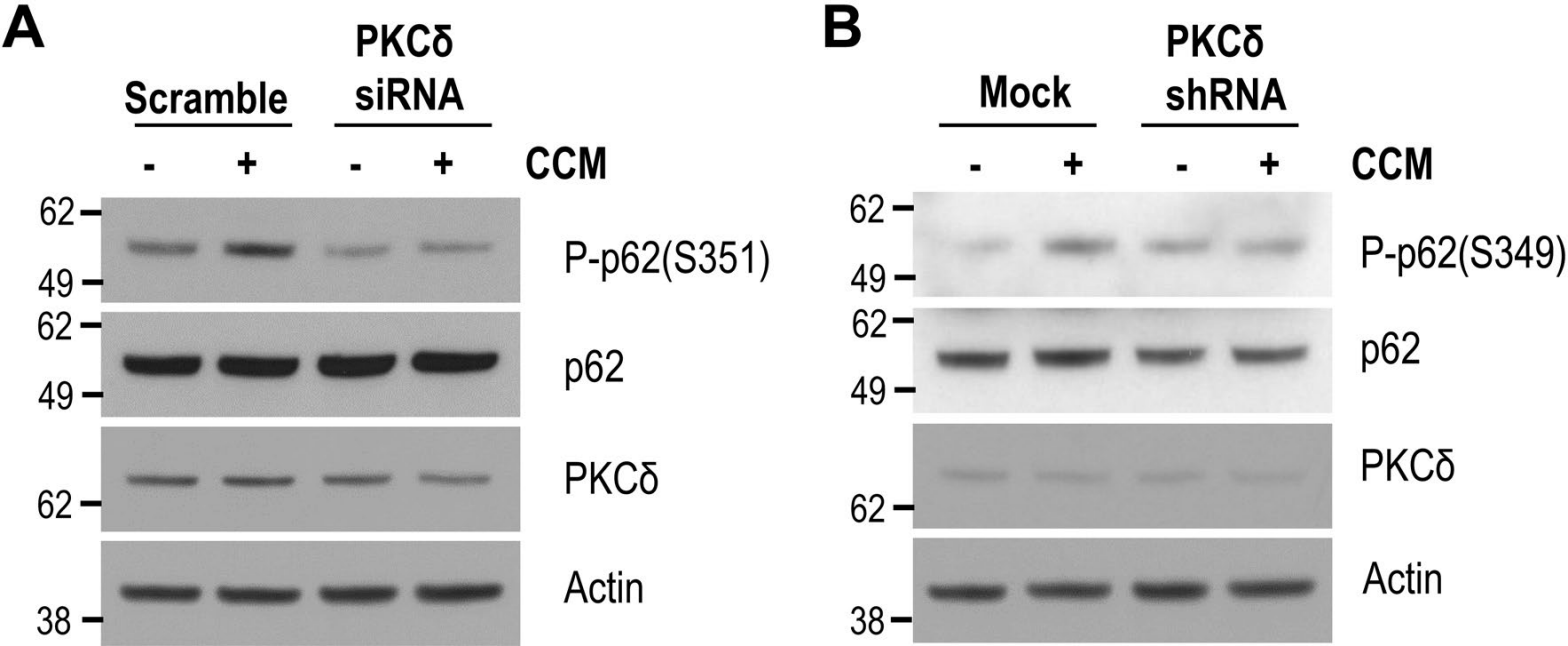
PKCδ is involved in p62 phosphorylation by curcumin. (**A**) Mouse cortical cells were transiently transfected with *PKCδ*-specific siRNA or scramble RNA as a control. The cells were treated with DMSO (−) or 10 μM curcumin ( +) for 12 h. The levels of phosphorylated p62 (S351), p62, PKCδ, and actin proteins were examined by immunoblotting using anti-phospho p62 (S349), p62, PKCδ, and actin antibodies, respectively. (**B**) HEK293 cells were transiently transfected with *PKCδ*-specific shRNA expressing plasmid or pcDNA3.1 (Mock), and treated with DMSO (−) or 10 μM curcumin (+) for 12 h. The levels of phosphorylated p62 (S349), p62, PKCδ, and actin proteins were examined by immunoblotting using anti-phospho p62 (S349), p62, PKCδ, and actin antibodies, respectively. Full blots are provided in Supplementary Fig. S11.

To determine whether PKCδ was involved in curcumin-induced phosphorylation of p62 at S351, we first investigated the activation of PKCδ using a T505 phospho-specific PKCδ antibody. As shown in Fig. 5A,B, PKCδ phosphorylation levels were increased in cells treated with curcumin, and peaked after 12 h of curcumin treatment, thereby indicating that PKCδ was activated in cells in the presence of curcumin. Notably, the amount of PKCδ which co-immunoprecipitated with p62 was also increased in cells treated with curcumin (Fig. 5C). Additionally, the level of p62 phosphorylation at S349 was enhanced in cells transfected with a PKCδ expression plasmid (Fig. 5D), but was diminished in cells with a *PKCδ*-specific shRNA expression plasmid (Fig. 6B). To confirm this, we pretreated neuronal cells with *PKCδ*-specific siRNA, and examined the level of p62 phosphorylation at S351 following curcumin treatment. As expected, p62 phosphorylation at S351 was abrogated in cells pretreated with *PKCδ*-specific siRNA (Fig. 6A). Taken together, these results strongly suggest that PKCδ is directly involved in the phosphorylation of p62 at S351 in the presence of curcumin.

### VPS34 is not involved in p62 phosphorylation at S351

**Figure 7:**
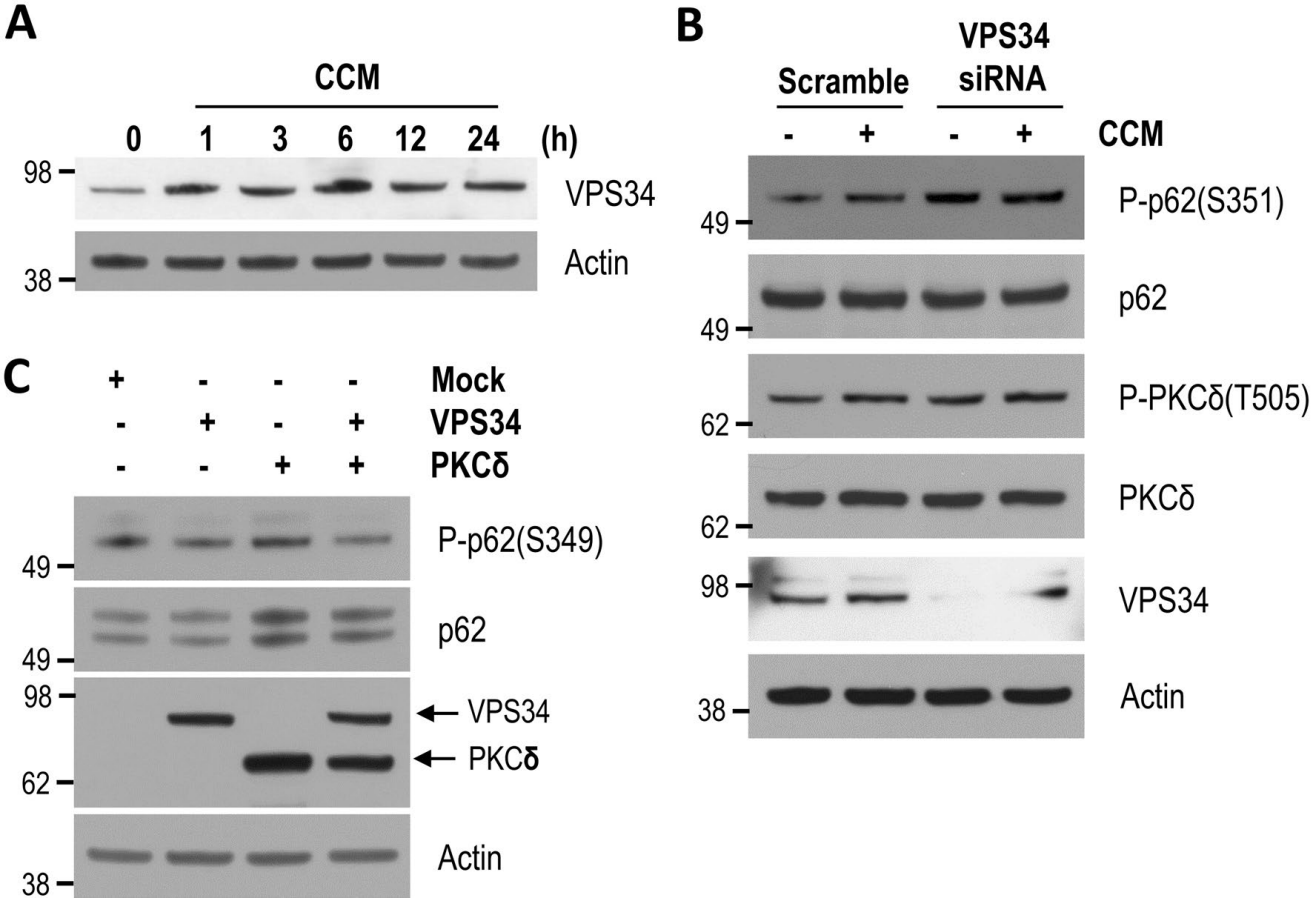
VPS34 is not involved in p62 phosphorylation by curcumin. (**A**) Mouse cortical cells were treated with DMSO (0 h) or 10 μM curcumin (CCM) for the indicated times. The protein levels of VPS34 and actin were analyzed by immunoblotting using anti-VPS34 and actin antibodies, respectively. (**B**) Mouse cortical cells were transiently transfected with *VPS34*-specific siRNA or scramble RNA as a control, and treated with DMSO (−) or 10 μM curcumin (+) for 12 h. The levels of phosphorylated p62 (S351), p62, VPS34, phosphorylated PKCδ (T505), PKCδ, and actin proteins were analyzed by immunoblotting using anti-phospho p62 (S349), p62, VPS34, phospho PKCδ (T505), PKCδ, and actin antibodies, respectively. (**C**) HEK293 cells were transiently transfected with HA-VPS34 and HA-PKCδ expression plasmids. The levels of phosphorylated p62 (S349), p62, VPS34, PKCδ and actin proteins were analyzed by immunoblotting using anti-phosphorylated p62 (S349), p62, HA and actin antibodies, respectively. Full blots are provided in Supplementary Fig. S11.

A previous study has suggested that VPS34 induces PKCδ-dependent phosphorylation of p62 at S351^24^. Therefore, we examined whether VPS34 was also involved in curcumin-induced p62 phosphorylation at S351 via PKCδ. To accomplish this, we determined the protein level of VPS34 in neuronal cells following curcumin treatment. The expression level of VPS34 in neuronal cells increased in the presence of curcumin (Fig. 7A). Unexpectedly, however, curcumin-mediated phosphorylation of p62 at S351 was not inhibited, but increased by transfection with *VPS34*-specific siRNA (Fig. 7B). Considering our results shown in Figs. 5 and 6, it could be expected that the increase of phosphorylated p62 at S351 in the cells treated with *VPS34*-specific siRNA might be resulted from an increased activity of PKCδ. To confirm it, we examined phosphorylation levels of PKCδ at T505 (active form) in the cells treated with *VPS34*-specific siRNA. As a result, any significant change in the phosphorylation levels of PKCδ in the cells treated with *VPS34*-specific siRNA was not observed compared to those in cells treated with scramble siRNA as a control, suggesting that PKCδ may be not involved in the increase of p62 phosphorylaton level by VPS34 knockdown (Fig. 7B). Additionally, p62 phosphorylation at S351 was induced in cells transfected with PKCδ, but the phosphorylation level was abated by co-expression of VPS34 (Fig. 7C). Altogether, these results demonstrate that curcumin induces PKCδ-mediated p62 phosphorylation at S351 but in a VPS34-independent manner.

## Discussion

**Figure 8:**
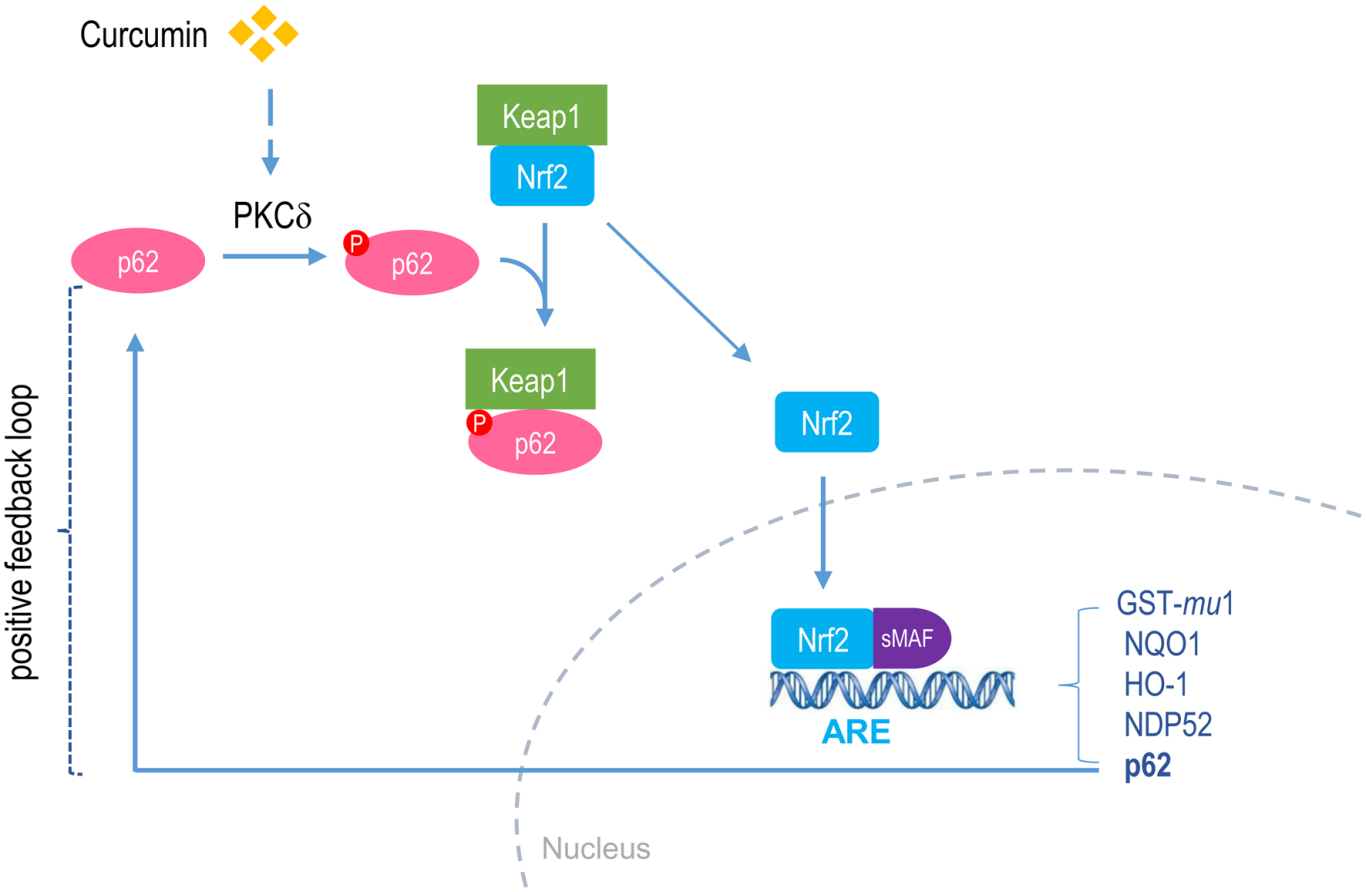
Schematic diagram showing the mechanism of Nrf2 activation by curcumin. Curcumin induces the phosphorylation of p62 at S351 by PKCδ activation. Phosphorylated p62 interferes the association of Nrf2 with Keap1, stabilizing Nrf2. Accumulated Nrf2 moves into nucleus and induces the expression of its downstream genes such as GST-*mu*1, NQO1, HO1, and p62 etc. Increased p62 takes part in the stabilization of Nrf2 again, thus forming a positive-feedback loop.

Nrf2 can be targeted pharmacologically in a variety of diseases, including neurodegenerative, vascular, and metabolic diseases underlined by oxidative stress and inflammation^28,29^. In the last two decades, many organic compounds have been developed as Nrf2 activators or inhibitors, some of which are under clinical trials for their therapeutic effect on different chronic diseases^16^. Among them is the natural compound curcumin, an electrophilic Nrf2 inducer and a ROS scavenger^30–32^. However, the exact mechanism by which curcumin activates Nrf2 remains to be explored. In the present study, we provide the first evidence that curcumin activates Nrf2 via PKCδ-mediated p62 phosphorylation at S351, which would inhibit the ability of Keap1 to trap Nrf2, resulting in Nrf2-stabilization and activation. Given that Nrf2 also induces the expression of p62 through transcriptional activation^25^, we speculate that Nrf2 activation by curcumin would be further enhanced by this positive feedback loop (Fig. 8).

PKCδ is ubiquitously expressed and involved in numerous signal transduction pathways related to cell proliferation and differentiation, inflammation, and apoptosis^33,34^. Various stimuli lead to PKCδ activation through phosphorylation or proteolytic cleavage to an active fragment^35,36^. It has been shown that PKCδ requires phosphorylation at S643, S662, and T505 for its full activation^37^, and of these three sites, phosphorylation at S662 and T505 is mediated by PKCζ or mTOR and PDK1, respectively^37–39^. We found that curcumin increased the level of PDK1 phosphorylation at S241 with a similar time point as when PKCδ is phosphorylated at T505 (Supplementary Fig. 5A). Therefore, it seems that curcumin-activated PDK1 may participate in the activation of PKCδ, leading to the phosphorylation of p62 at S351. This notion is consistent with that of a previous study which suggests that curcumin activates Nrf2 in human monocytes via PKCδ^40^. Indeed, our data showed that curcumin induced the activation of PKCδ in different cells. Additionally, PKCδ is known to directly phosphorylate Nrf2 at S40, which is required for the dissociation of Nrf2 from Keap1^41^. Therefore, it is expected that curcumin-mediated PKCδ activation not only phosphorylates p62 at S351, but also phosphorylates Nrf2 at S40, thus cooperatively enhancing the dissociation of Nrf2 from Keap1.

In addition to PKCδ, several kinases such as mTORC1, CK1, and Tak1 have been shown to participate in the phosphorylation of p62 at S351 under normal cellular conditions^21,23,24^. We found that, in neuronal cells treated with curcumin, the level of p62 phosphorylation at S351 was not changed by pretreatment with (5Z)-7-Oxozeanol, a Tak1 inhibitor, and CKI7, a CK1 inhibitor. In contrast, the level of p62 phosphorylation at S351 was attenuated in the presence of mTORC1 inhibitors such as PP242 or Torin1 (Supplementary Fig. 3), suggesting that mTORC1 might be involved in p62 phosphorylation. However, accumulating evidence suggests that curcumin suppresses mTORC1 activation^42,43^. In this study, there was no significant change in the phosphorylation levels of ULK1 at S757, a substrate of mTORC1 and of mTORC1 at S2448, in cells treated with curcumin, compared with untreated control cells (Supplementary Fig. 4). Thus, we postulate that phosphorylation of p62 at S351 occurs continuously through the basal activity of mTORC1, rather than via induction by curcumin. However, given that PKCδ directly phosphorylates p62 at S351 (Figs. 5, 6), it is possible that the phosphorylation of p62 at S351 may be marginally inhibited by mTORC1 inhibitors because the phosphorylation of PKCδ at S662 by mTORC1 is necessary for its full activation as previously described^37^.

VPS34, a class III lipid kinase generating PI(3)P, is essential for protein sorting to the vacuole via the endolysosomal pathway^44,45^. Interestingly, a previous study suggested that VPS34 promotes the association of PKCδ with p62, thus enhancing the phosphorylation of p62 at S351^24^. Here, we observed increased expression of VPS34 protein following curcumin treatment (Fig. 7A); however, pretreatment of neuronal cells with SAR405, a VPS34-specific inhibitor, did not affect curcumin-induced phosphorylation of p62 at S351 (Supplementary Fig. 3). Additionally, ectopic expression of VPS34 and knockdown of VPS34 using a *VPS34*-specific siRNA reduced and augmented the level of p62 phosphorylation, respectively (Fig. 7B,C). Intriguingly, VPS34 likely plays a negative regulator in curcumin-induced p62 phosphorylation at S351 according to our results (Fig. 7). Our findings suggest that PKCδ-mediated phosphorylation of p62 at S351 is not dependent on VPS34, in contrast to the previous report^24^.

p62 undergoes extensive post-translational modifications including phosphorylation, and interacts with various signaling molecules, thereby allowing fine-tuned regulation of its function^17^. The phosphorylation of p62 at S351 increases its binding affinity with Keap1, thereby activating Nrf2 via a non-canonical mechanism^21,22^. Importantly, the p62 S351 is phosphorylated in selective autophagy conditions such as impaired proteostasis, mitochondrial depolarization, and bacterial infection^21,46,47^. In a previous study, we observed increased phosphorylation of p62 at S351 in addition to Nrf2 activation in TFEB-expressing cells^48^. Thus, the Keap1-Nrf2 pathway and selective autophagy, the major stress response pathways, are coupled to each other via the phosphorylation of p62^17^. We also observed that the addition of curcumin to neuronal cells markedly induced p62 phosphorylation at S403 in its ubiquitin-associated (UBA) domain, which is known to increase the affinity between UBA and polyubiquitin chain^49^ (data not shown). Consequently, p62 associated with Keap1 via the P-STGE motif is expected to be translocated to the ubiquitinated cargos in the presence of curcumin. Then, Nrf2 free from Keap1 is accumulated in the cells, thus resulting in Nrf2-activation. To further prove that PKCδ is directly involved in p62 phosphorylation at S351, we pretreated neuronal cells with various PKCδ chemical inhibitors including rottlerin, bisindolylmaleimide I, and Gӧ6983 etc., before curcumin treatment. Unexpectedly, the phosphorylation level of p62 at S351 was highly increased with only treatment of PKCδ inhibitors to the cells. Given that rottlerin is well known as a strong uncoupler^50^, the effect was comprehensive, but not in cases of other inhibitors. PKC inhibitors have a wide spectrum of inhibitory activity for PKCs; therefore, it seems that the inhibition of PKC signaling raises some stress to cells, and then resulting in p62 phosphorylation at S351. The precise mechanism remains to be elusive.

Nrf2 activity is tightly controlled at several steps via its ubiquitination and proteasomal degradation. To date, the following three types of E3 ubiquitin ligases have been reported to participate in Nrf2 degradation by the proteasome, independently of Keap1: β-TrCP (β-transducin repeat-containing protein)^51^, DCAF11 (DDB1 and Cullin4 associated factor 11, also referred to as WDR23)^52^, and Hrd1 (HMG-CoA reductase degradation 1 homolog, also referred to as synoviolin)^53^. β-TrCP interacts with the Neh6 domain phosphorylated by GSK-3β, while DCAF11 binds to the DIDLID sequence of the Neh2 domain in Nrf2^52,54^. Some research groups have suggested that the PI3K-AKT signaling pathway is involved in curcumin-induced Nrf2 activation^6,31^. Since the phosphorylation of AKT and GSK-3β was increased in neuronal cells treated with curcumin (Supplementary Fig. 5A), we speculate that β-TrCP-mediated Nrf2 degradation may be inhibited by the decreased activity of GSK-3β. However, cells pretreated with AKT inhibitor IV, surprisingly exhibited higher levels of Nrf2 protein and p62 phosphorylation at S351 (Supplementary Fig. 5B). Also, the increased level of Nrf2 in the presence of curcumin was not ameliorated by transfection of constitutively active GSK-3β (Supplementary Fig. 6). Thus, it seems that the PI3K-AKT/GSK-3β signaling pathway is, if any, partially involved in Nrf2 activation by curcumin. Interestingly, in the late-stage following curcumin treatment, the level of β-TrCP protein dramatically increased, whereas that of Nrf2 decreased (Supplementary Fig. 8), thereby indicating that β-TrCP played a critical role in the termination of curcumin-induced Nrf2 activation by promoting Nrf2 degradation.

Although curcumin has been known to be passed through the blood brain barrier^4,5^, there are two imitations: poor solubility in water and low permeability^55^. In fact, about 96% of oral curcumin is excreted in the feces and urine after 72 h in rats^56^. Interestingly, by a single oral administration of 5 mg curcumin in mice, approximately 5 ng of curcumin in one gram brain tissue was detected after 8 h, corresponding to 13.5 nM^57^. We observed that 1 μM curcumin was able to significantly increase Nrf2 protein level and ARE promoter activity (above 60%) (Supplementary Fig. 7). Here, we used 10 μM curcumin to investigate whether it activates Nrf2, enhancing the expression of its down-stream genes. Even though the permeability could be different according to species, our study supports that taking 5 g of curcumin via the oral route could lead to 13.5 μM curcumin in brains. Recently, to increase the transport efficiency of curcumin to the brain, a variety of formulations have been developed^58^, which is expected to help to increase its possibility in successful prevention and therapeutic use for brain diseases.

Abundant preclinical and clinical evidence indicates that curcumin has potential as a therapy for various chronic diseases including neurodegenerative and inflammatory diseases^2^. Unlike other drugs, it can impact a diverse range of targets and signaling pathways^2,12^. To date, several studies on the activation of Nrf2 by curcumin have been reported, but the exact mechanism of activation remains controversial. Here, we have proposed a novel, non-canonical mechanism whereby curcumin activates Nrf2 via p62 phosphorylation at S351 through PKCδ activation. Thus, our results provide a scientific clue to understand the biological activity of curcumin in cells and to develop this natural compound as a therapeutic drug for chronic diseases, including neurodegenerative diseases.

## Methods

### Antibodies, reagents, and plasmids

Anti-Nrf2 (12721), NDP52 (9036), phospho p62 (S349, 16177), phospho PKCδ (T505, 9374), PKCδ (9616), VPS34 (4263) and Myc (9B11, 2276), HA (6E2, 2367) antibodies were purchased from Cell Signaling Technology. Anti-GSTM1 (SC-133641) antibody was obtained from Santa Cruz Biotechnology. Anti-Nrf2 rat monoclonal antibody (14596) was purchased from Cell Signaling Technology. Anti-heme oxygenase (HO)-1 (ADI-SPA-895) and p62 (BML-PW9860) antibodies were purchased from Enzo Life Sciences. Anti-NQO1 (11451-1-AP) and Keap1 (10503-2-AP) antibodies were purchased from Proteintech. Anti-β-actin (MAB1501) antibody was obtained from Millipore. Curcumin was purchased from Calbiochem. Protease inhibitor cocktail and other chemicals were purchased from Sigma-Aldrich. The plasmids expressing Myc-Nrf2, Myc-p62 and Myc-p62 (S349A), and containing 3xARE-Luc reporter gene were used as described in previous studies^48,59,60^. *PKCδ* and *VPS34*-specific siRNA was purchased from Bioneer (Korea) and Dharmacon, respectively. Plasmids expressing VPS34, PKCδ, and *PKCδ-*specific shRNA were obtained from Addgene.

### Cell culture

Cells were cultured as described in the previous studies^48,59^**.** Immortalized mouse cortical neuronal cells were cultured in the Dulbecco’s modified Eagle medium (DMEM) supplemented with 10% heat-inactivated fetal bovine serum (FBS), and 10 units/ml penicillin and 100 units/ml streptomycin in a humidified atmosphere of 95% air and 5% CO_2_ at 33 °C. Wild-type and *p62* gene knockout (−/−) mouse embryonic fibroblasts (MEFs) were gifted by Dr. Cho (Kyungpook National Univ., Korea). MEFs and HEK293 cells were maintained in DMEM supplemented with 10% heat-inactivated FBS, 10 units/ml penicillin, and 100 units/ml streptomycin in a humidified atmosphere of 95% air and 5% CO_2_ at 37 °C.

### Transient transfection and luciferase assay

Cells were transiently transfected with the relevant plasmids or siRNA using Lipofectamine 2000 (Thermo Fisher Scientific) according to the manufacturer’s instructions. The total amount of DNA or siRNA used for each well was normalized to the relevant mock vectors or scramble RNA. For the luciferase assay, the cells were transiently co-transfected with the ARE-Luc and TK-Renilla plasmids using Lipofectamine 2000. Luciferase and Renilla activities were measured using the Luciferase Assay System (E2920, Promega) and the GloMax 20/20 Luminometer (Promega) as previously described in the study^48^. Relative luciferase activity was calculated by normalizing to that of Renilla activity.

### Immunoblotting

Cells were washed once with PBS and lysed with modified RIPA buffer (10 mM Tris–HCl [pH 7.4], 150 mM NaCl, 1 mM EGTA, 1% NP-40, 0.25% sodium deoxycholate, 0.1% SDS) containing 1 mM NaF, 1 mM Na_3_VO_4_, and 1× protease inhibitor cocktail as previously described in the study^48^. Proteins were extracted on ice by periodic vortexing for 30 min. Lysates were cleared by centrifugation at 12,000 rpm for 10 min at 4 °C, and the supernatants were used for immunoblotting following boiling in 1× SDS-sample loading buffer and 1 × reducing agent for 7 min. For analysis, protein samples (20 μg) were separated on NuPAGE 4–12% Bis–Tris gels (Invitrogen) at a constant current of 20 mA, followed by transfer to nitrocellulose membranes (GE Healthcare). The membranes were incubated with the indicated antibodies overnight at 4 °C following blocking with 5% skim milk in TBST (20 mM Tris–HCl [pH 7.4], 500 mM NaCl, and 0.5% Tween-20). Blots were washed with TBST, and then incubated with horseradish peroxidase-linked secondary antibody for 1 h at room temperature. Protein bands were developed with chemiluminescence (Thermo Fisher Scientific) following washing with TBST. All protein concentrations were determined using the BCA method (Sigma-Aldrich).

### Immunohistochemical staining

Immunohistochemical staining was performed as described in the previous study^48^. Mouse cortical cells were fixed on coverslips with 4% paraformaldehyde for 30 min. After cell permeabilization, the cells were incubated with a blocking solution (2% normal goat serum, 0.1% Triton-X-100 in PBS) for 1 h. After briefly washing with PBS, the cells were probed with rat anti-Nrf2 (1:250) antibody diluted in the blocking solution at 4 °C overnight. Then, the cells were incubated with donkey anti-rat Alexa488 conjugated antibody (1:500) for 1 h at room temperature. The coverslips were mounted on glass slides with the ProLong Gold Antifade Reagent (P36935, Invitrogen) and analyzed by the Olympus FV1000 laser scanning confocal microscope.

### Quantitative real-time PCR

For cDNA synthesis, total RNA was extracted from cells using the RNeasy Mini Kit (Qiagen) according to the manufacturer’s instructions. cDNA was synthesized from 2 μg of RNA using RT-PCR EcoDry Premix (Clontech). Quantitative real-time PCR (qRT-PCR) was performed using the SYBR Green Real-Time PCR protocol (4344463, Invitrogen) on the QuantStudio 6 Flex Real-Time PCR System (Applied Biosystems) as previously described in the study^48^. Each reaction consisted of 10 μL of 35-fold diluted cDNA, 2.5 µM of each primer (mouse Nrf2, 5′-GGCTCAGCACCTTGTATCTT-3′ and 5′-CACATTGCCATCTCTGGTTTG-3′; mouse HO-1, 5′-GTACACATCCAAGCCGAGAA-3′ and 5′-TGGTACAAGGAAGCCATCAC-3′; mouse GSTM1, 5′-GACTTTCCCAATCTGCCTTACT-3′ and 5′-CTCCTCCTCTGTCTCTCCATC-3′; mouse p62, 5′-GTGGTGGGAACTCGCTATAAG-3′ and 5′-GAAAGATGAGCTTGCTGTGTTC-3′; mouse NDP52, 5′-AGACCCTGACGAGGACATAA-3′ and 5′-AGGTCAGCGTACTTGTCTTTC-3′; mouse NQO1, 5′-GAGAAGAGCCCTGATTGTACTG-3′ and 5′-ACCTCCCATCCTCTCTTCTT-3′; mouse GAPDH, 5′-TCAACAGCAACTCCCACTCTTCCA-3′ and 5′-ACCCTGTTGCTGTAGCCGTATTCA-3′), and 12.5 µL of SYBR Green Real-Time PCR Master Mix (Applied Biosystems) in a total volume of 25 µL. The reactions were incubated in a 96-well plate at 95 °C for 10 min, followed by 40 cycles of 95 °C for 15 s and 60 °C for 1 min. After the reactions were completed, the threshold was manually set and the cycle threshold (CT) was automatically recorded. The relative mRNA levels were calculated by CT values, which were normalized to GAPDH mRNA. All reactions were performed in triplicate for each sample.

### Measurement of endogenous ROS

ROS was measured as previously described in the study^48^. Mouse cortical cells were treated with curcumin for 6 h followed by the incubation with 10 μM 6-carboxy-2′,7′-dichlorodihydrofluorescein diacetate (DCF-DA, Sigma-Aldrich) in DMEM without serum for 30 min. Then, the cells were rinsed twice with PBS. For flow cytometry analysis, the cells were detached with trypsin and resuspended in PBS. Endogenous ROS was measured by the DCF fluorescence using the FITC channel on the BD FACSVerse System (BD Biosciences).

## Supplementary Information

Supplementary Information.
