# Supplementary material for: Curcumin activates Nrf2 through PKCδ-mediated p62 phosphorylation at Ser351
Source: Sci Rep. 2021 Apr 19;11:8430. doi: 10.1038/s41598-021-87225-8 (PMC8055680; doi:10.1038/s41598-021-87225-8)
Supplement: Supplementary file 1 — Supplementary Information. [file 41598_2021_87225_MOESM1_ESM.pdf]

# Curcumin activates Nrf2 through PKCδ-mediated p62 phosphorylation at Ser351

Jee-Yun Park[1], Hee Young Sohn[1], Young Ho Koh[1], and Chulman Jo[1*]

[1]Division of Brain Disease Research, Department for Chronic Disease Convergence Research, Korea National Institute of Health, 187 Osongsaengmyeong2-ro, Osong-eup, Cheongju-si, Chungcheongbuk-do 363-951, Korea

*Correspondence and requests for materials should be addressed to C.J.

E-mail: chulmanjo@gmail.com

Phone: +82-43-719-8631

# Supporting Information

## Methods

**Antibodies, and reagents.** Anti-phospho mTOR (S2448, 5536), mTOR (2983), phospho ULK1 (S757, 14202), ULK1 (8054), phospho PDK1 (S241, 3438), PDK1 (5662), phospho AKT (S473, 4060), AKT (4691), phospho GSK-3β (S9, 5558), GSK-3β (9315) and β-TrCP (4394) antibodies were purchased from Cell Signaling Technology. Anti-LC3 (PD014) and lamin A/C (sc-20681) antibodies were obtained from MBL and Santa Cruz Biotechnology, respectively. Anti-tau (A0024) and tubulin (T6074) antibodies were purchased from Dako and Sigma, respectively. Torin1 (475991) were purchased from Calbiochem. SB203580 (1202), SP600125 (1496), CKI7 dihydrochloride (5329), (5Z)-7-Oxozeaenol (3604), PP242 (4257), and wortmannin (1232) were obtained from Tocris. AKT inhibitor IV (sc-203809) and U0126 (BPS-27012) were obtained from Santa Cruz and Biomol, respectively. The plasmids expressing GSK-3β (S9A) and human tau protein were described in the previous study[1]. Anti-PHF1 antibody specific for tau phosphorylated at S396/404 was used in the previous study[2].

**Cell viability assay.** Mouse cortical cells were treated with curcumin of varying concentrations (0-20 μM) for 24 h. The spent media were replaced with fresh media containing either 250 μg/ml thiazolyl blue tetrazolium bromide (MTT, Sigma) or 0.5 mg/mL resazurin (Sigma), and then incubated for 1 h at 37°C in a humidified atmosphere containing 5% $CO_2$. The MTT formazan crystals were dissolved in 200 μL of DMSO. Absorbance was measured at 570 nm wavelength using a SpectraMax 190 microplate reader (Molecular Devices). For the resazurin assay, the fluorescence intensity was detected using a SpectraMax M4 microplate reader (Molecular Devices) at an excitation wavelength of 540 nm and an emission wavelength of 535 nm.

**Preparation of nuclear and cytosolic fractions.** Mouse cortical neuronal cells were washed with and scraped in PBS. Cell pellets were resuspended in fractionation buffer (10 mM HEPES [pH 7.9], 10 mM KCl, 1.5 mM $MgCl_2$, 0.1% NP-40, 0.5 mM NaF, 200 mM $Na_3VO_4$ and 1 × protease inhibitor cocktail). The cells were incubated on ice for 15 min with shaking. Lysates were centrifuged at 2,600 × g at 4°C, and supernatants representing cytosolic fraction were collected. Subsequent to washing precipitates using the fractionation buffer without 0.1% NP-40, the precipitates then were resuspended with the modified RIPA buffer containing 1x protease inhibitor cocktail and incubated on ice for 20 min with periodic vortexing. The lysates were then cleared by centrifugation at 10,000 × g at 4°C, and supernatants were used as the nuclear fractions.

## References


1. Kim, S. *et al.* Fisetin stimulates autophagic degradation of phosphorylated tau via the activation of TFEB and Nrf2 transcription factors. *Scientific reports* **6**, 24933, doi:10.1038/srep24933 (2016).
2. Jo, C. *et al.* Nrf2 reduces levels of phosphorylated tau protein by inducing autophagy adaptor protein NDP52. *Nature communications* **5**, 3496, doi:10.1038/ncomms4496 (2014).

Supplementary Fig. 1

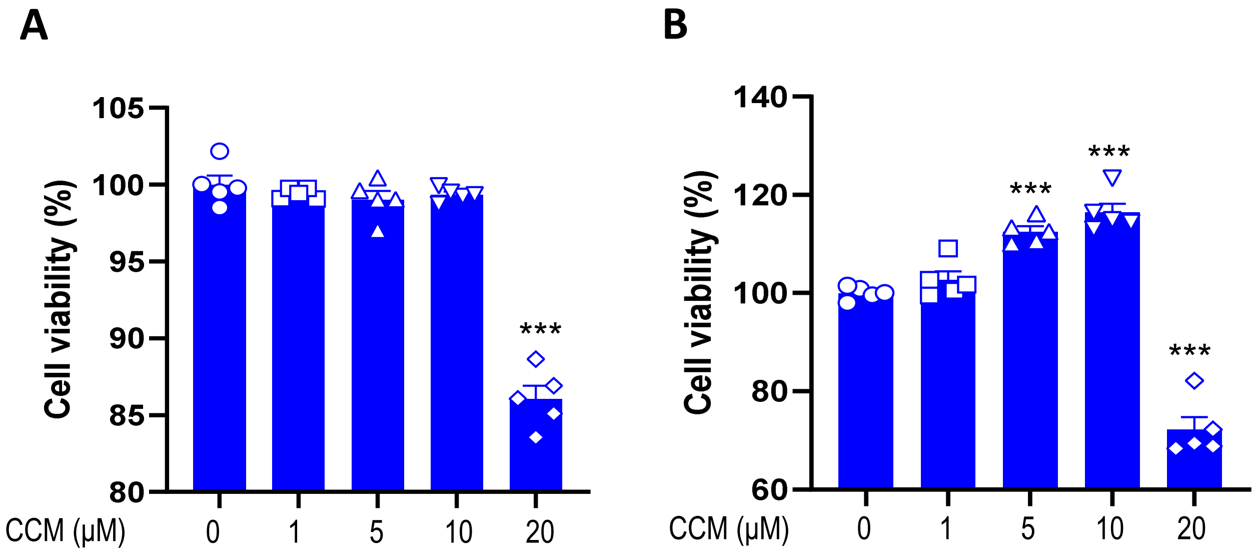


**S1 Fig. Curcumin does not decrease cell viability at doses up to 10 μM**. Mouse cortical cells were treated with DMSO (0) or a range of concentration of curcumin (CCM, 1-20 μM) for 24 h. Cell viability was detected by MTT (**A**) and resazurin assays (**B**). Data shown are mean ± S.E. of three independent experiments and were analyzed using the Student's *t*-test. (*$^{***}p<0.001$*)

Supplementary Fig. 2

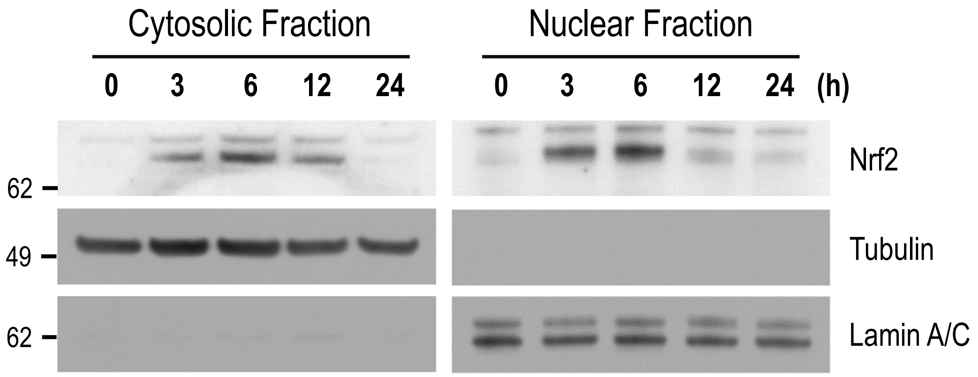


**S2 Fig. Curcumin increases the protein level of Nrf2 and induces its nuclear localization.** Mouse cortical cells were treated with 10 μM curcumin for the indicated times. Cytosolic and nuclear fractions from the cells were prepared according to the procedure described in Methods. The cellular localization of Nrf2 was analyzed by immunoblotting using an anti-Nrf2 antibody. To examine the purity of the fractionations, the blot was probed with antibodies to lamin (A/C), a marker for nuclear fraction, and tubulin, a marker for cytosolic fraction. Full blots are provided in Supplementary Fig. 11.

Supplementary Fig. 3

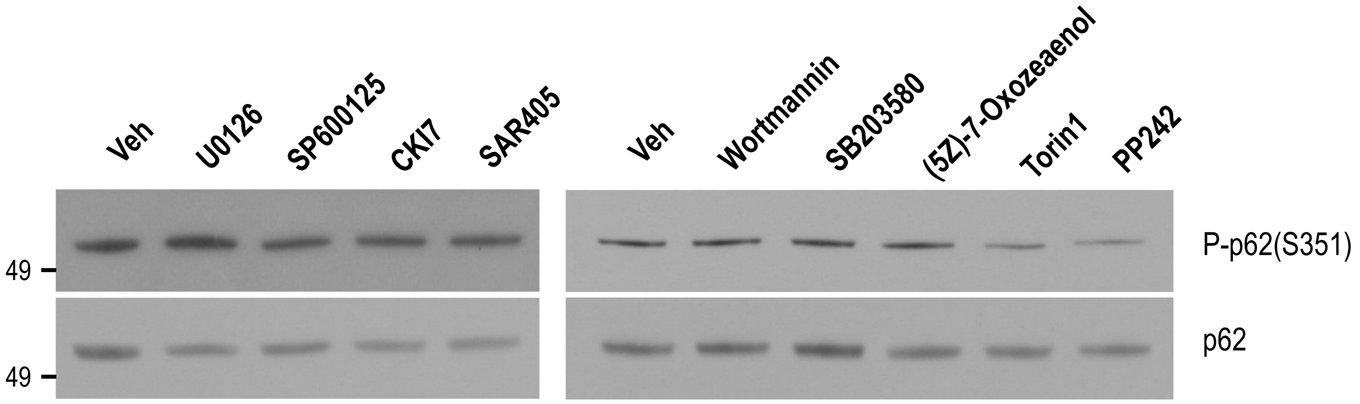


**S3 Fig. mTORC1 inhibition decreases curcumin-induced p62 phosphorylation at S351**. Mouse cortical cells were pre-treated with DMSO (Veh) or the following inhibitors for 30 min: 5 μM U0126 (ERK inhibitor), 10 μM SP600125 (JNK inhibitor), 100 μM CKI7-dihydrochloride (CKI7, CK1 inhibitor), 5 μM SAR405 (VPS34 inhibitor), 1 μM wortmannin (PI3K inhibitor), 20 μM SB203580 (p38 inhibitor), 250 nM (5Z)-7-Oxozeaenol (TAK1 inhibitor), 250 nM Torin1 (mTORC1 inhibitor) and 1 μM PP242 (mTORC1 inhibitor). The cells were treated with 10 μM curcumin for 12 h, the levels of phosphorylated p62 (S351) and p62 proteins were analyzed by immunoblotting using anti-phospho p62 (S349) and p62 antibodies, respectively. Full blots are provided in Supplementary Fig. 11.

Supplementary Fig. 4

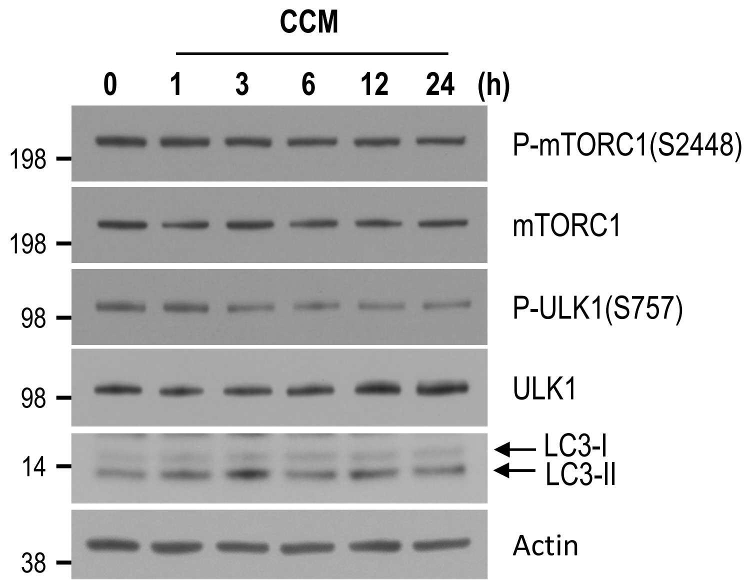


**S4 Fig. Curcumin does not activate mTORC1 signaling**. Mouse cortical cells were treated with DMSO (0 h) or 10 μM curcumin (CCM) for the indicated times. The levels of phosphorylated mTORC1 (S2448), mTORC1, phosphorylated ULK1 (S757), ULK1, LC3, and actin proteins were analyzed by immunoblotting using anti-phospho mTORC1 (S2448), mTORC1, phospho ULK1 (S757), ULK1, LC3, and actin antibodies, respectively. Full blots are provided in Supplementary Fig. 11.

Supplementary Fig. 5

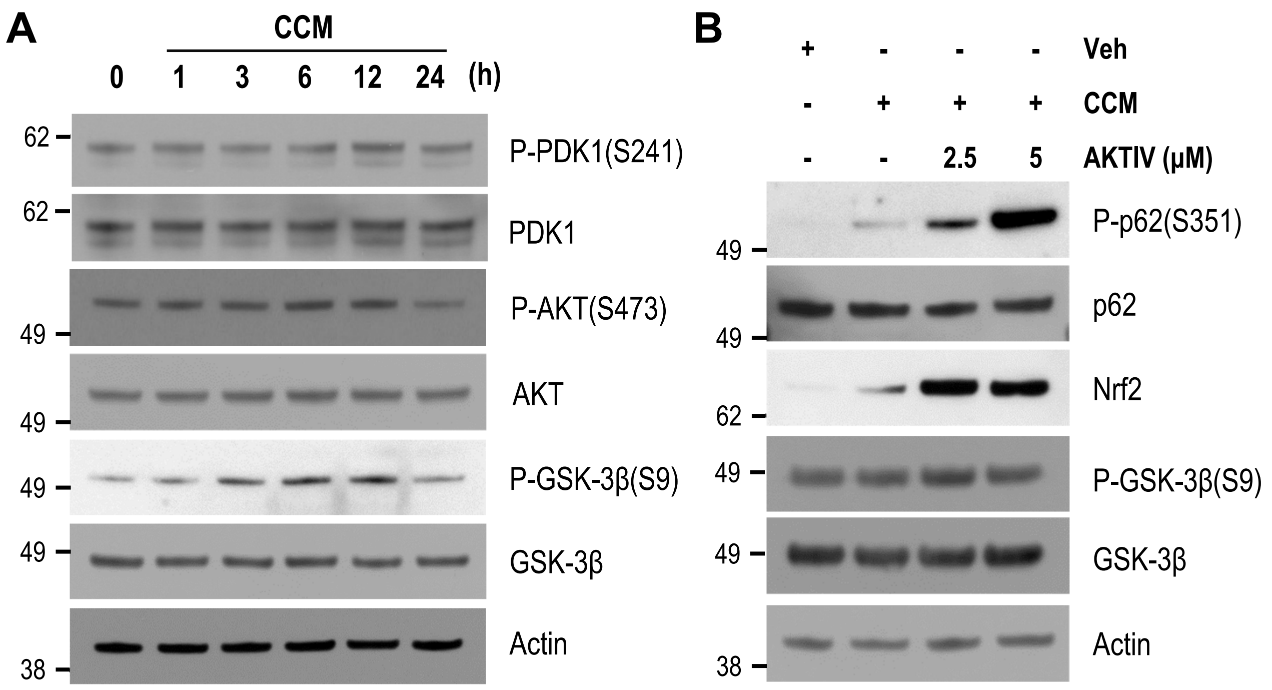


**S5 Fig. AKT is not involved in curcumin-mediated p62 phosphorylation at 351**. (**A**) Mouse cortical cells were treated with DMSO (0 h) or 10 μM curcumin (CCM) for the indicated times. The levels of phosphorylated PDK1 (S241), PDK1, phosphorylated AKT (S473), AKT, phosphorylated GSK-3β (S9), GSK-3β, and actin proteins were analyzed by immunoblotting using corresponding antibodies, respectively. (**B**) Mouse cortical cells were pre-treated with DMSO (Veh) or AKT inhibitor IV (AKTIV), and then were treated 10 μM curcumin (CCM) for 12 h. The levels of phosphorylated p62 (S351), p62, Nrf2, phosphorylated GSK-3β (S9), GSK-3β, and actin proteins were analyzed by immunoblotting using corresponding antibodies, respectively. Full blots are provided in Supplementary Fig. 11.

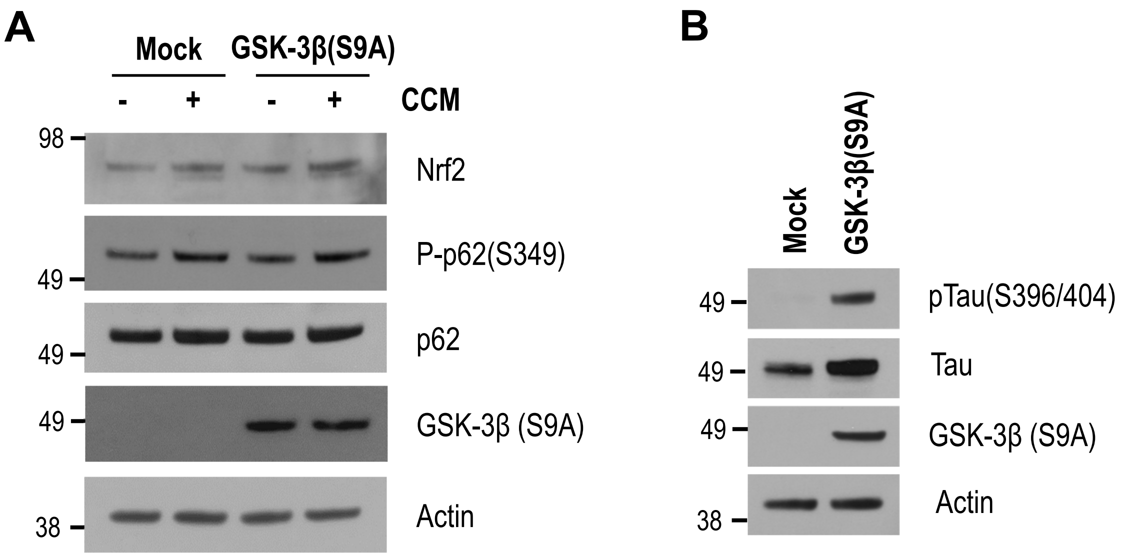


**S6 Fig. GSK-3β is not likely to be mainly involved in curcumin-induced Nrf2 activation**. (A) HEK 293 cells were transiently transfected with the plasmid expressing constitutively active HA-tagged GSK-3β (S9A), and treated with DMSO (0 h) or 10 μM curcumin (CCM) for 12 h. The levels of Nrf2, phosphorylated p62 (S349), p62, GSK-3β (S9A), and actin proteins were analyzed by immunoblotting using corresponding antibodies, respectively. (B) HEK 293 cells were transiently co-transfected with the plasmid expressing human tau protein along with pcDNA3.1 (Mock) or constitutively active HA-tagged GSK-3β (S9A). On the next day, the levels of phosphorylated tau (S396/404), total tau, GSK-3β (S9A), and actin proteins were analyzed by immunoblotting using anti-PHF1, tau, HA, and actin antibodies, respectively. Full blots are provided in Supplementary Fig. 11.

Supplementary Fig. 7

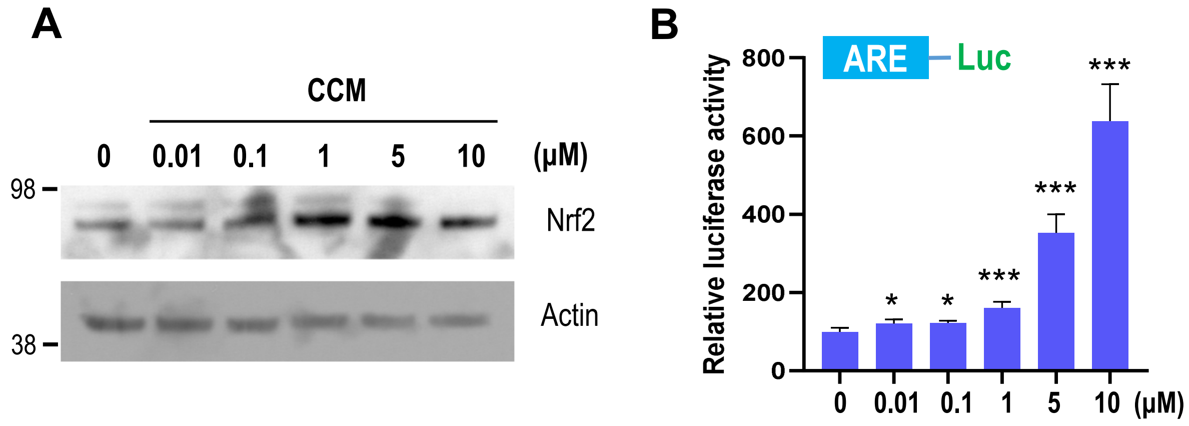


**S7 Fig. One μM of curcumin is able to significantly increase in Nrf2 protein level and promoter activity containing ARE elements.** (**A**) Mouse cortical cells were treated with DMSO (0) or a range of concentration of curcumin (0.01-10 μM) for 12 h. The levels of Nrf2, and actin proteins were analyzed by immunoblotting using corresponding antibodies, respectively. Full blots are provided in Supplementary Fig. 11. (**B**) HEK293 cells were transiently transfected with ARE-Luc reporter and TK-Renilla plasmids. After treatment with either DMSO (0) or a range of concentration of curcumin (0.01-10 μM) for 12 h, the cells were assayed for the luciferase activity. Data shown are mean ± S.E. of three independent experiments and were analyzed using the Student's *t*-test. (*$p<0.05$, ***$p<0.001$)

Supplementary Fig. 8

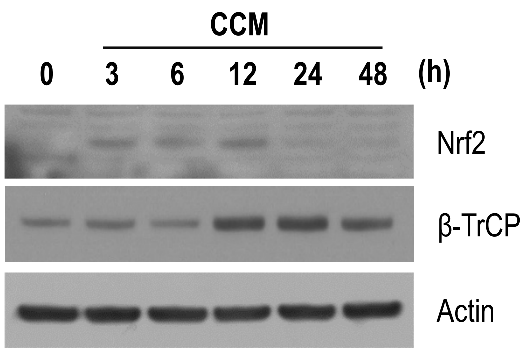


**S8 Fig. β−TrCP is dramatically induced in the late stage of Nrf2 activation**. Mouse cortical cells were treated with DMSO (0 h) or 10 μM curcumin (CCM) for the indicated times. The levels of Nrf2, β-TrCP, and actin proteins were proteins were analyzed by immunoblotting using corresponding antibodies, respectively Full blots are provided in Supplementary Fig. 11.

Supplementary Fig. 9

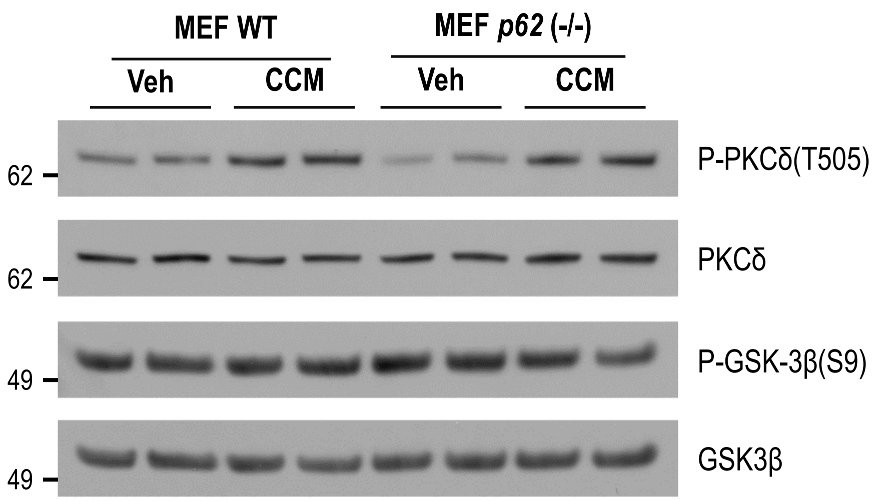


**S9 Fig. PKCδ is activated in curcumin-treated p62 knockout (-/-) MEF cells**. MEFs were treated with DMSO (Veh) or 10 μM curcumin (CCM) for 12 h. The protein levels of phosphorylated PKCδ (T505), PKCδ, phosphorylated GSK3β (S9), and GSK3β were analyzed by immunoblotting using corresponding antibodies, respectively. Full blots are provided in Supplementary Fig. 11.

Supplementary Fig. 10

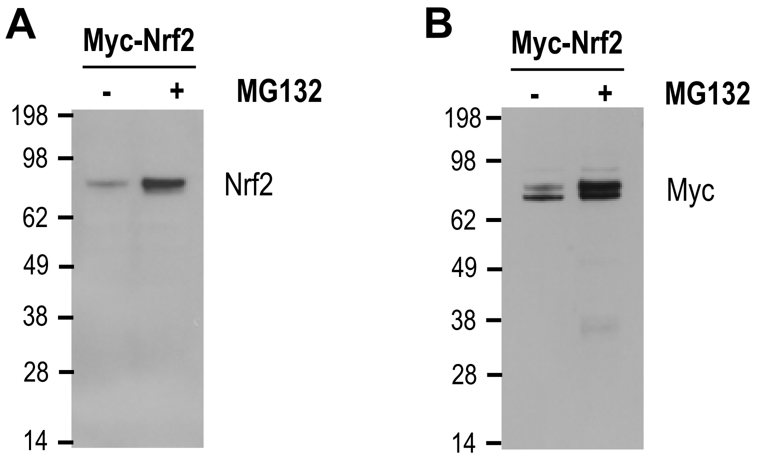


**S10 Fig. Nrf2 protein is around 80-90 kDa on gradient (4-12%) SDS-PAGE gels**. HEK293 cells were transiently transfected with a plasmid expressing Myc tagged-Nrf2. On the next day, half of cells were treated by proteasome inhibitor MG132 (5 μM) for 12 h. Then, protein samples (20 μg) were separated on NuPAGE® 4%-12% Bis-Tris gels (Invitrogen) at a constant current of 20 mA, followed by transfer to nitrocellulose membranes as described in Methods. Nrf2 protein was analyzed by immunoblotting using anti-Nrf2 (**A**) and anti-Myc (**B**) antibodies, respectively. Full blots are provided in Supplementary Fig. 11.

Supplementary Fig. 11

Fig. 1B

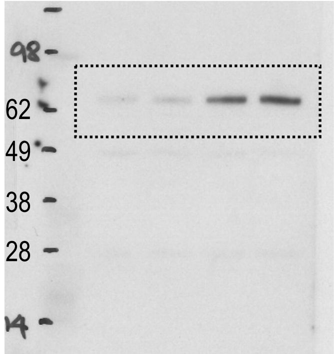


Nrf2

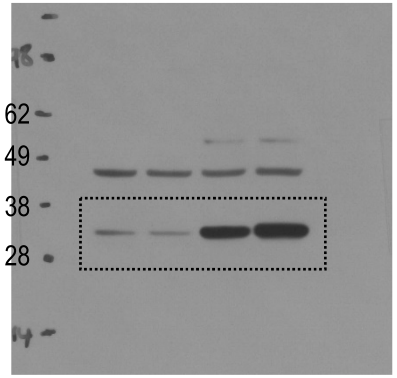


HO-1

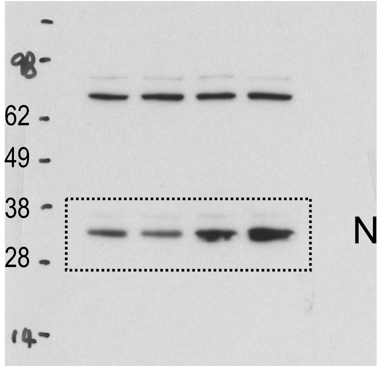


NDP52

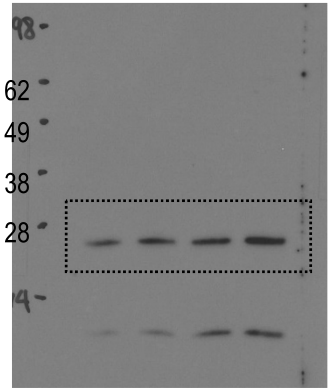


GST-*mu*1

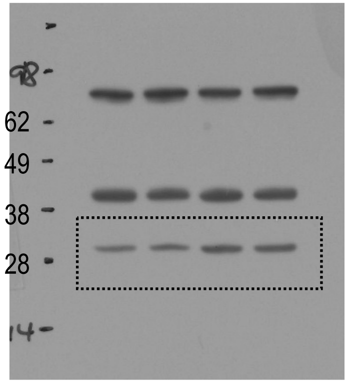


NQO1

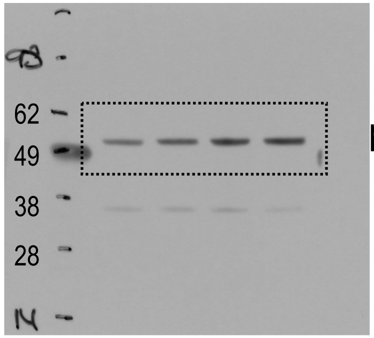


p62

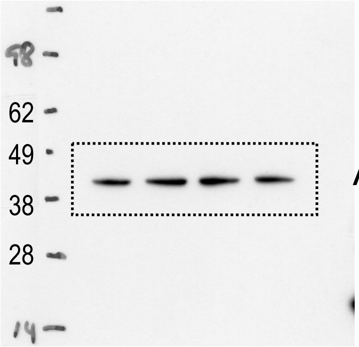


Actin

Supplementary Fig. 11 continued

Fig. 3A

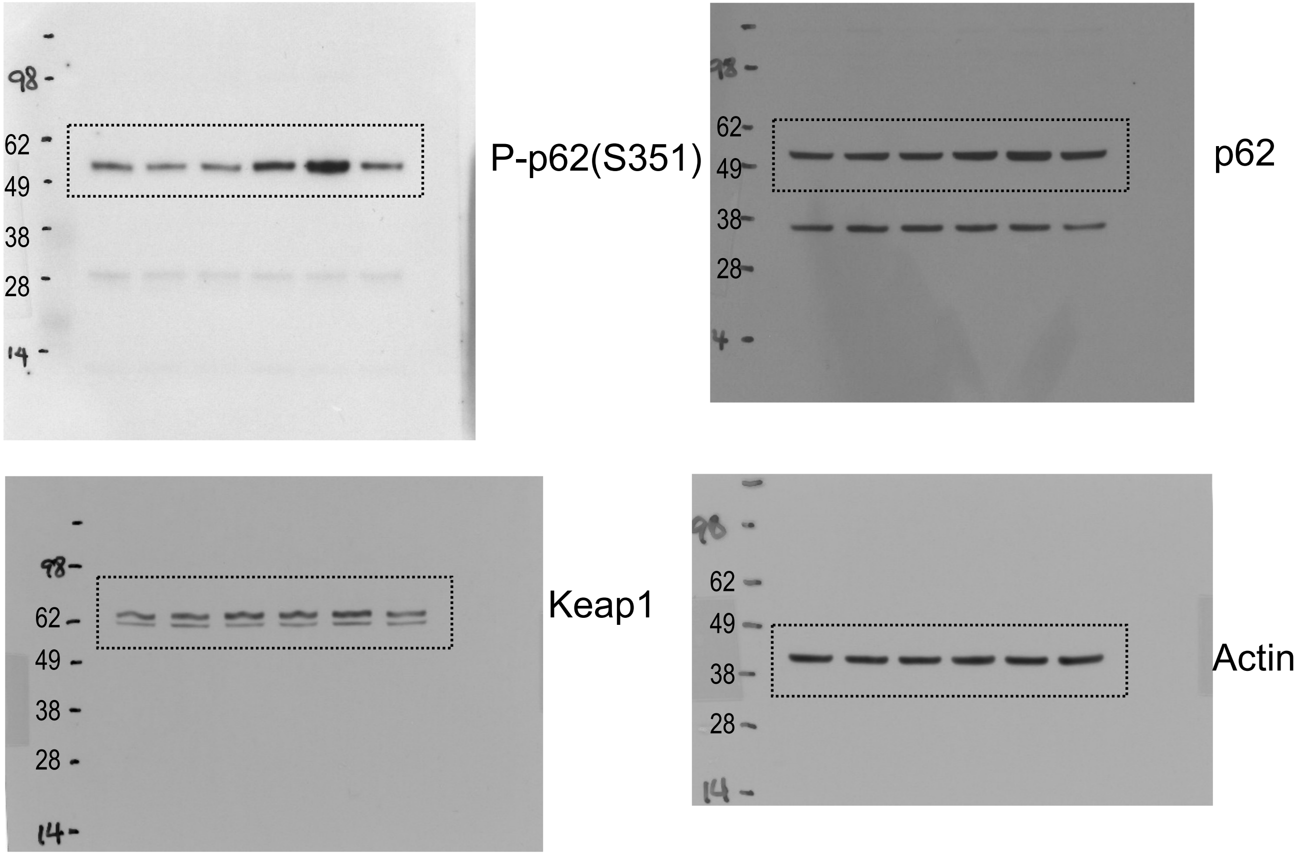


Supplementary Fig. 11 continued

Fig. 3B

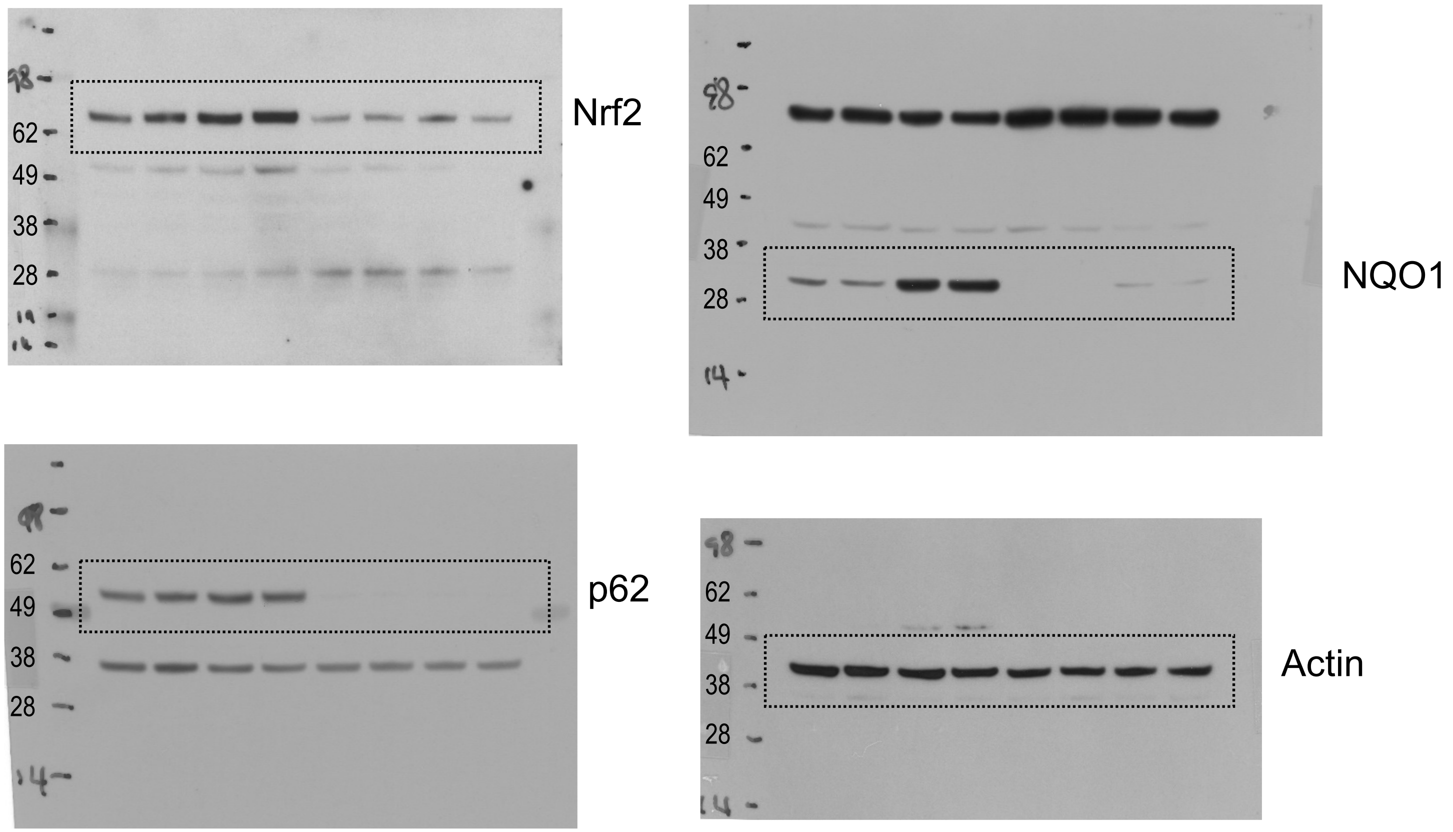


Supplementary Fig. 11 continued

Fig. 4A

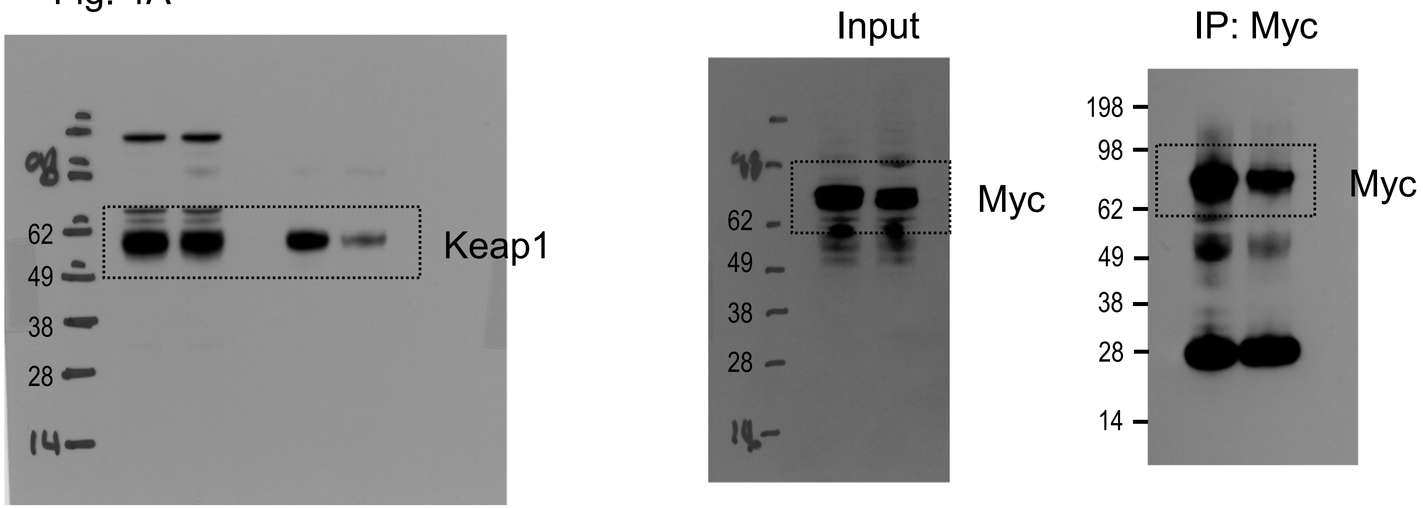


Supplementary Fig. 11 continued

Fig. 5A

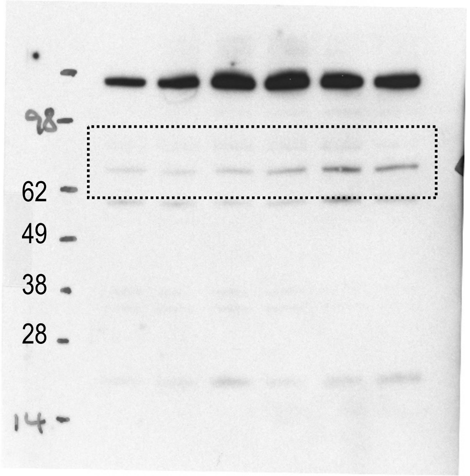


P-PKCδ(T505)

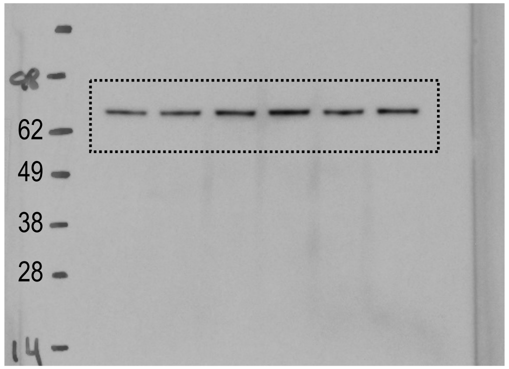


PKCδ

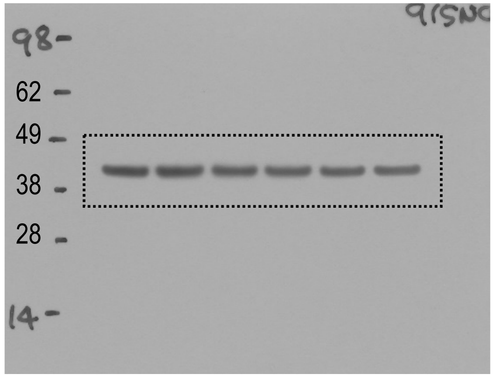


Actin

Supplementary Fig. 11 continued

Fig. 5B

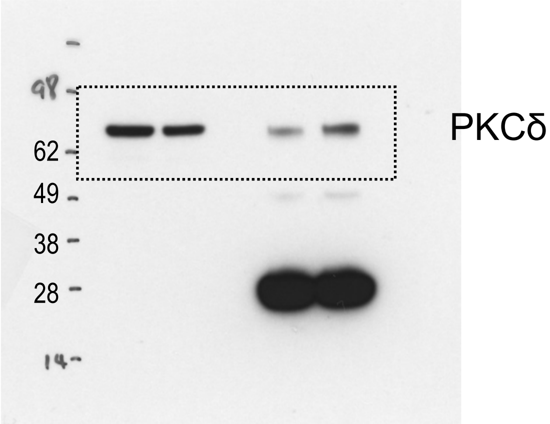


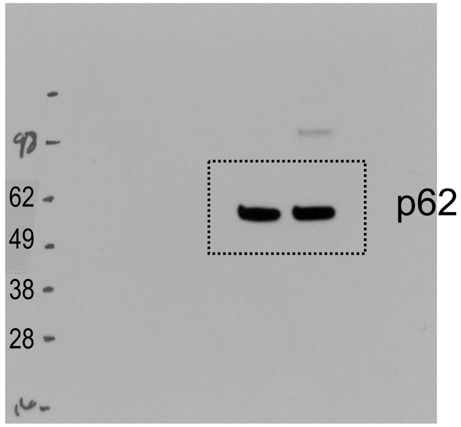


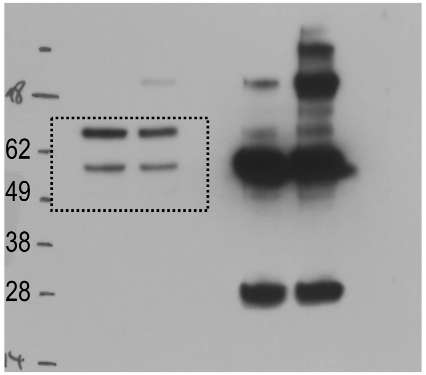


Supplementary Fig. 11 continued

Fig. 5D

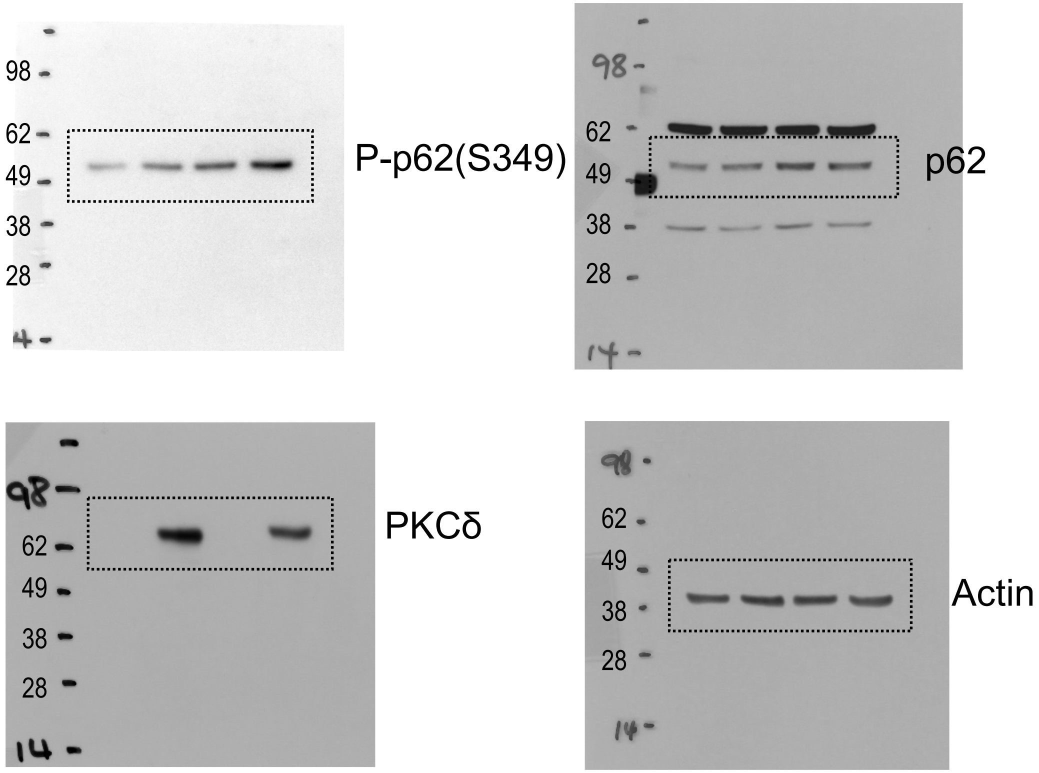


Supplementary Fig. 11 continued

Fig. 6A

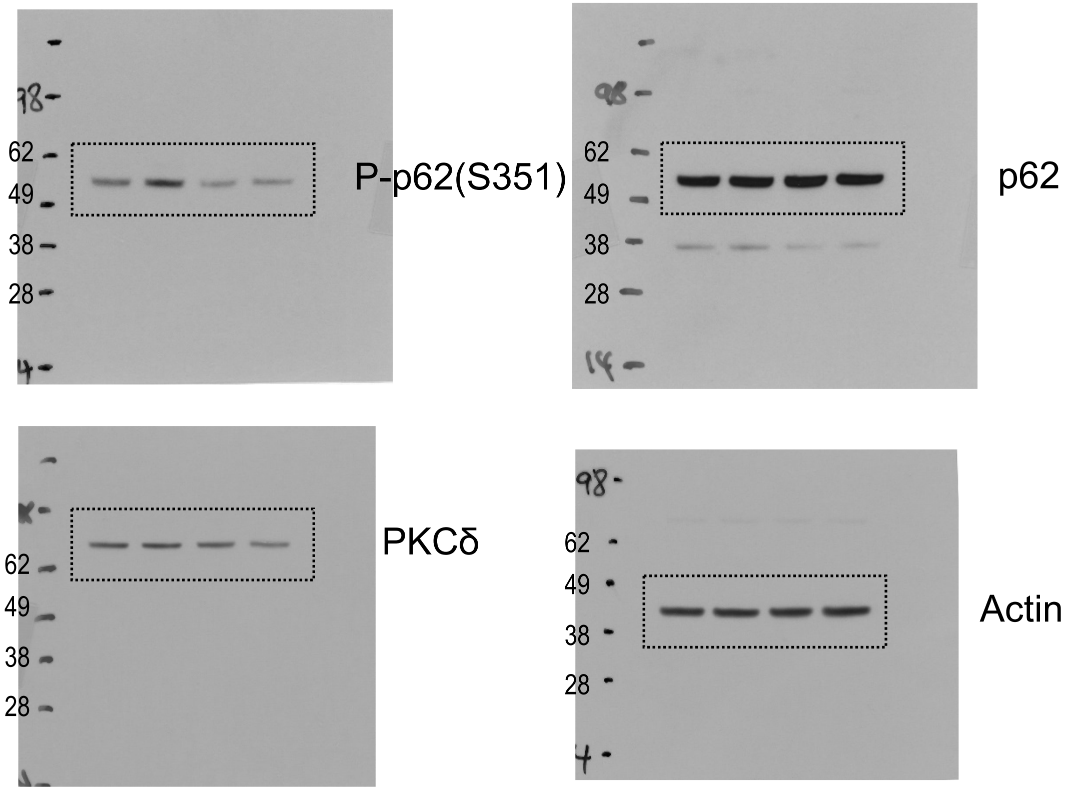


Supplementary Fig. 11 continued

Fig. 6B

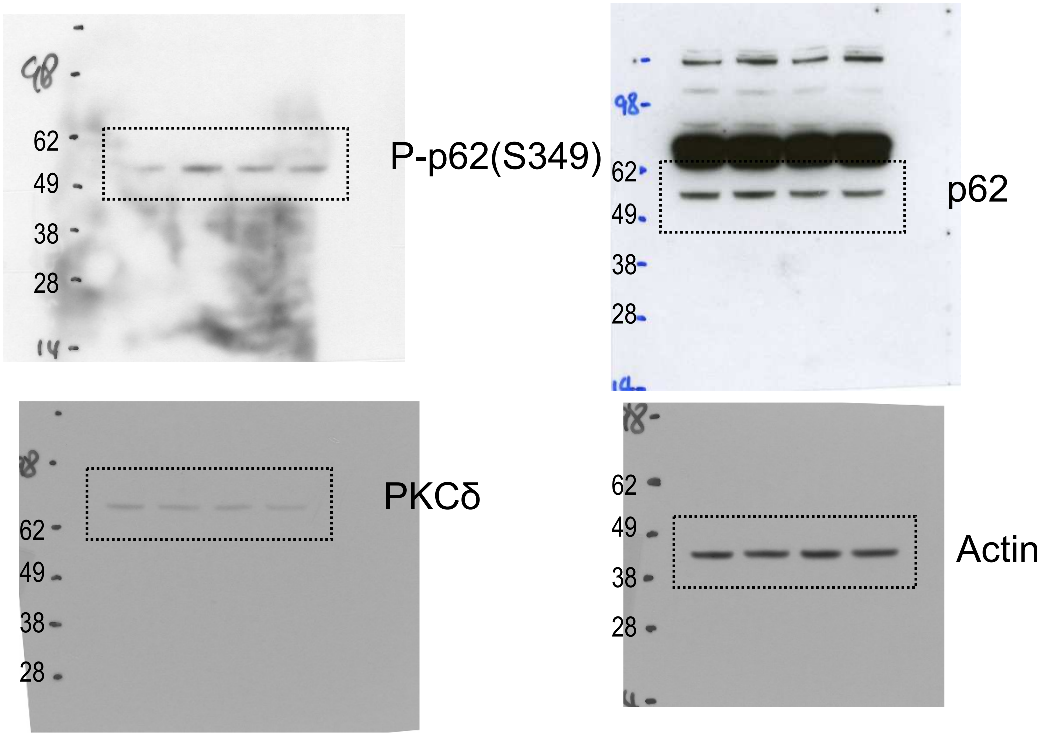


Supplementary Fig. 11 continued

Fig. 7A

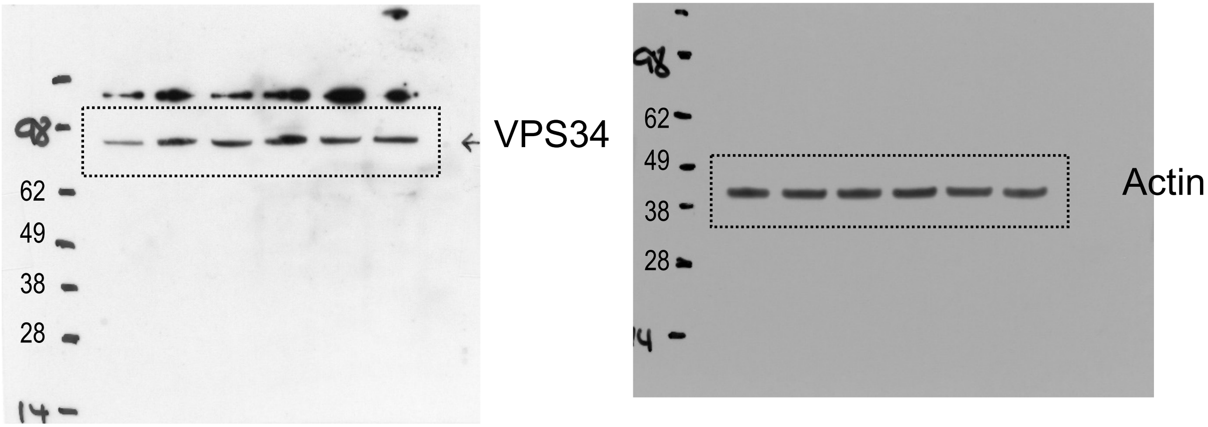


Supplementary Fig. 11 continued

Fig. 7B

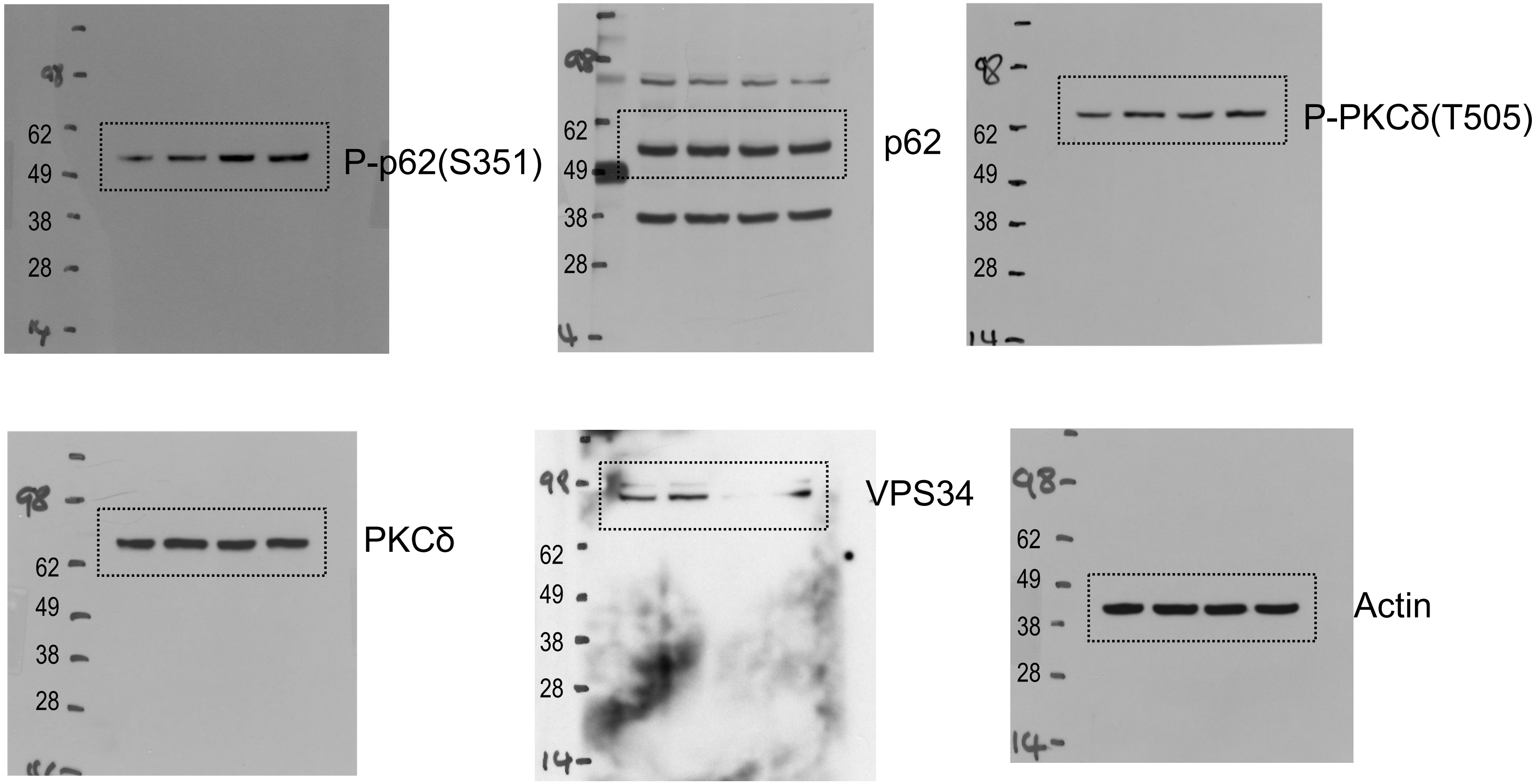


Supplementary Fig. 11 continued

Fig. 7C

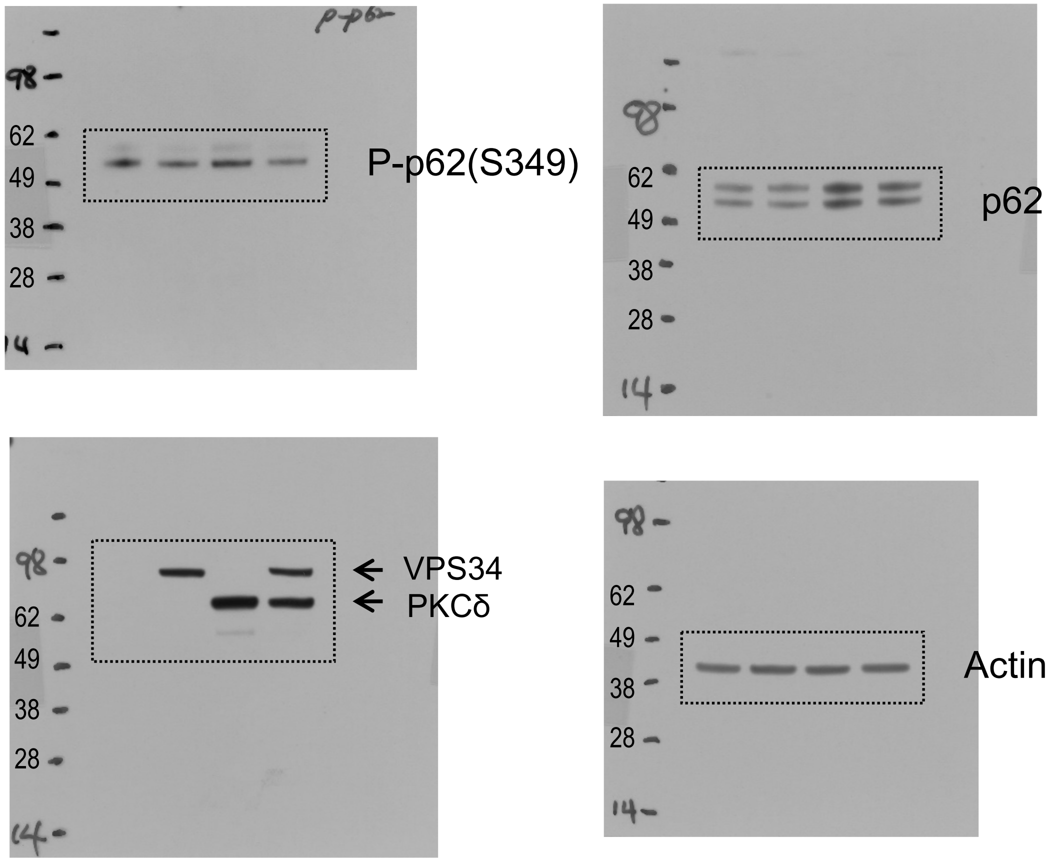


Supplementary Fig. 11 continued

Supplementary Fig. 2

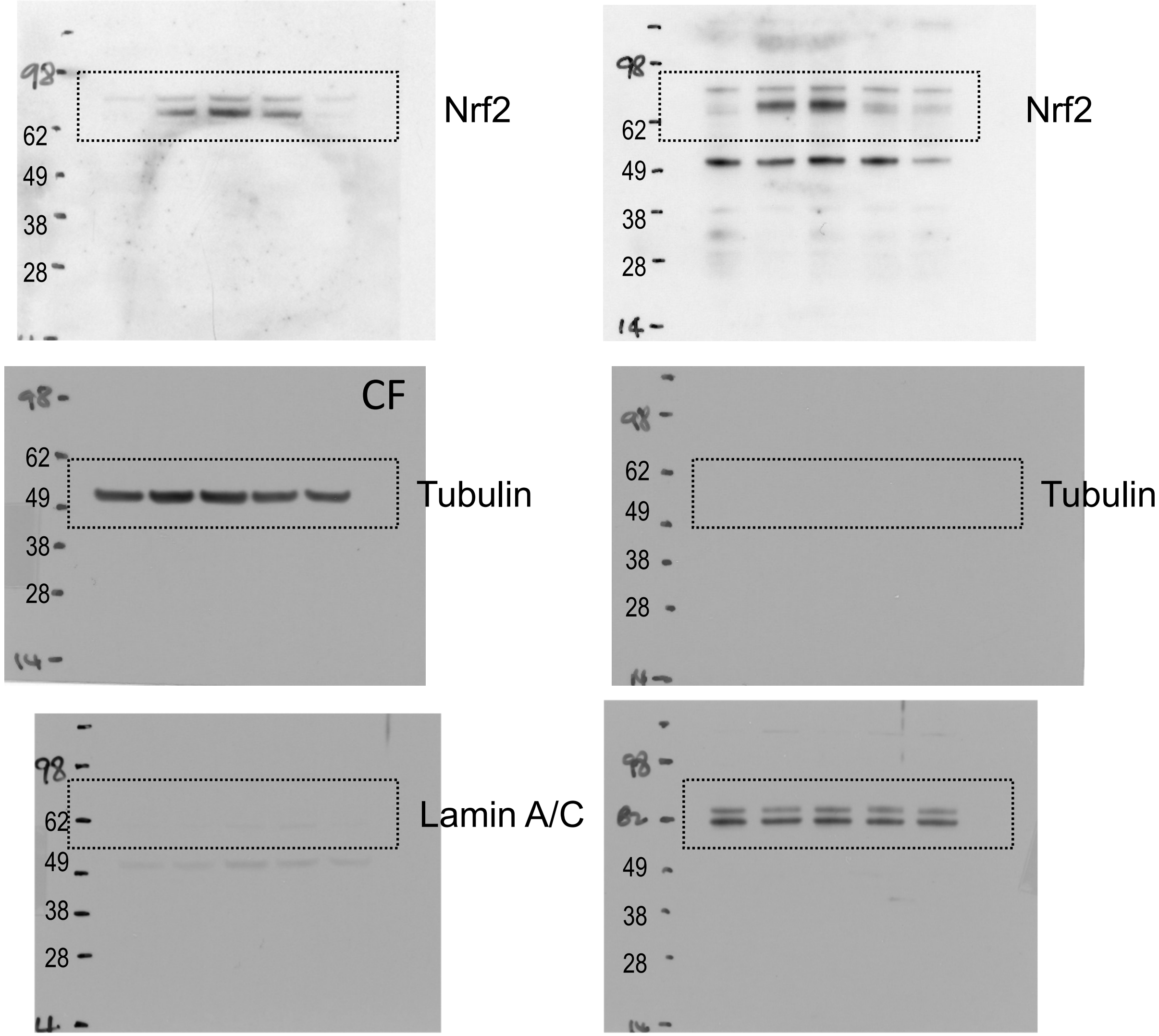


Supplementary Fig. 11 continued

Supplementary Fig. 3

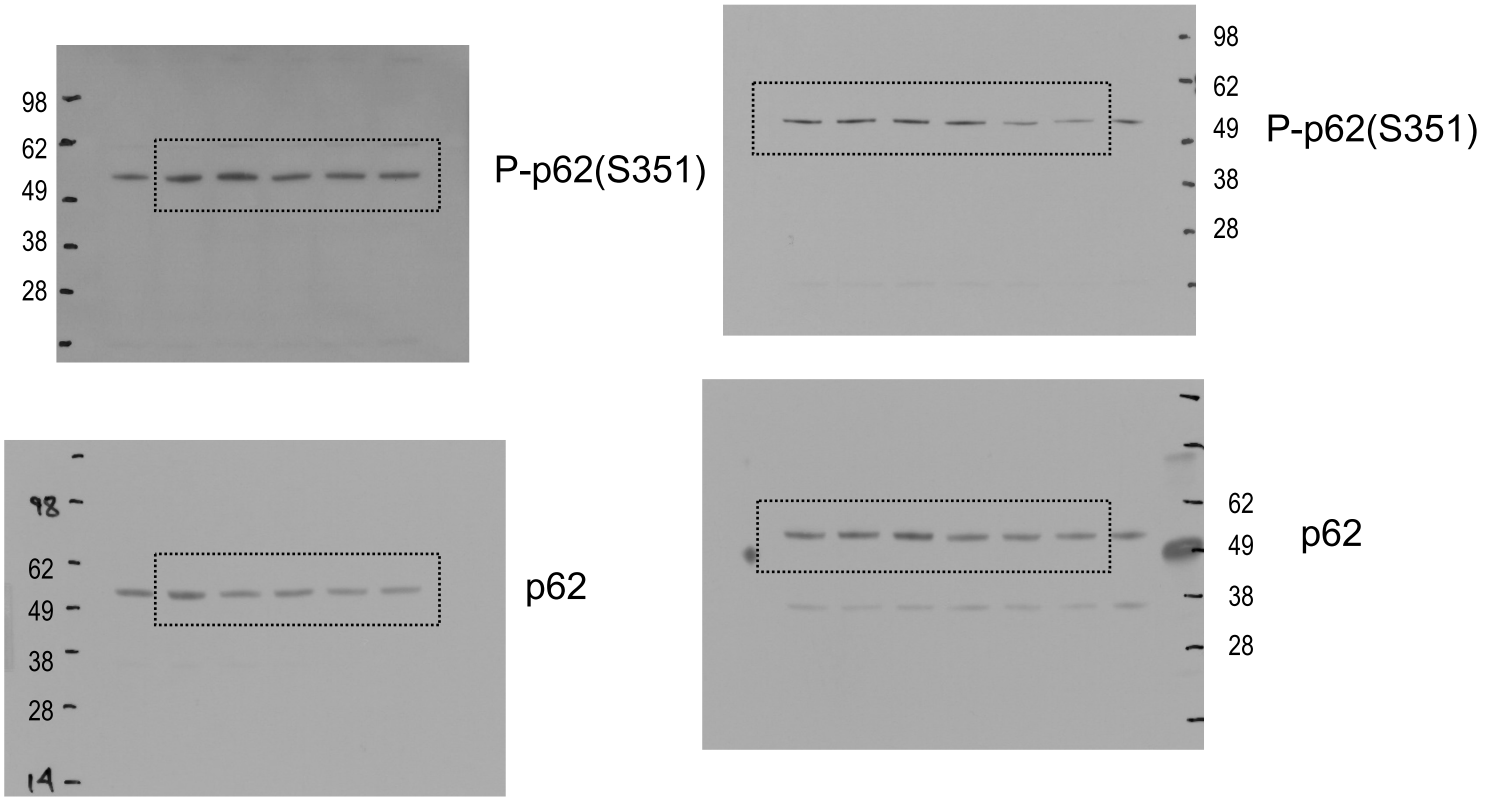


Supplementary Fig. 11 continued

Supplementary Fig. 4

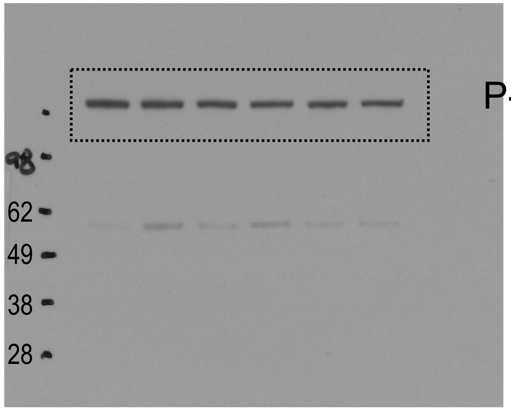


P-mTORC1(S2448)

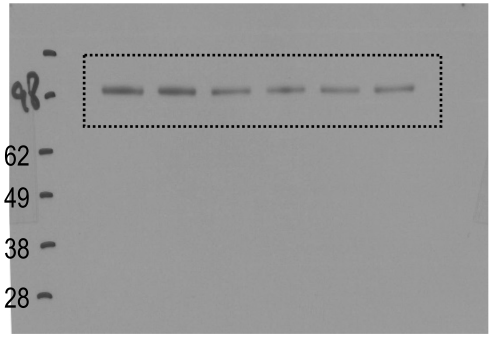


P-ULK1

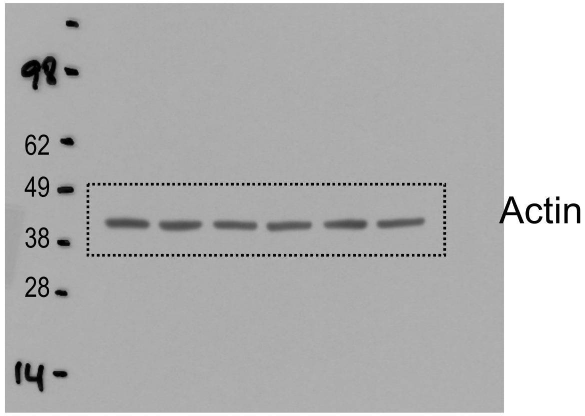


Actin

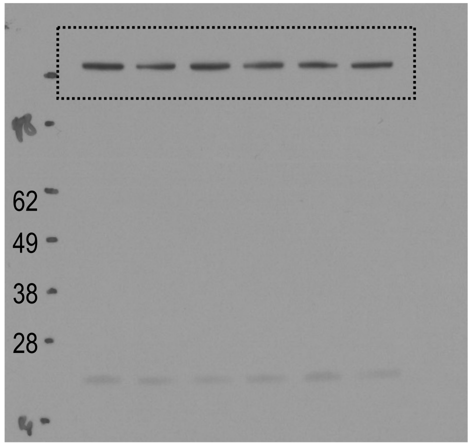


mTORC1

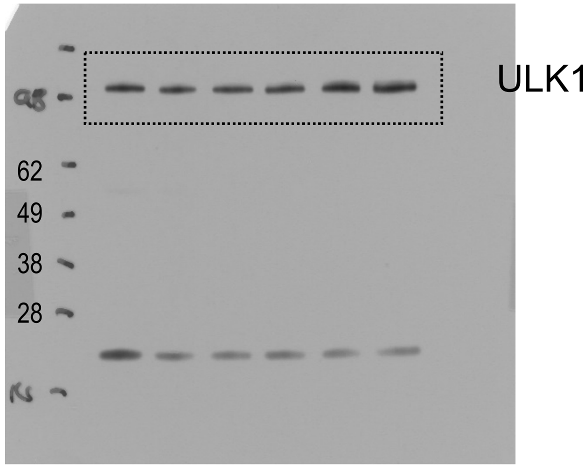


ULK1

Supplementary Fig. 11 continued

Supplementary Fig. 5A

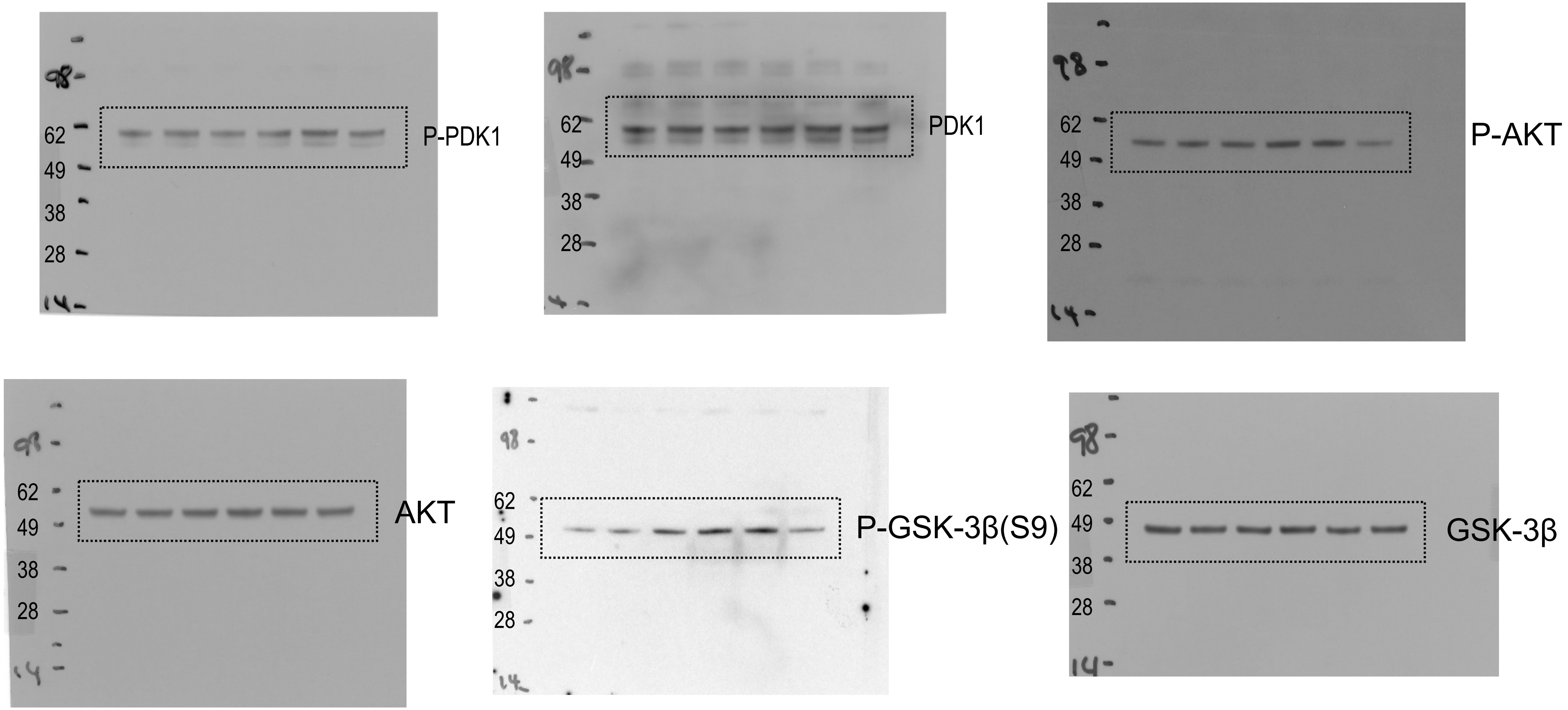


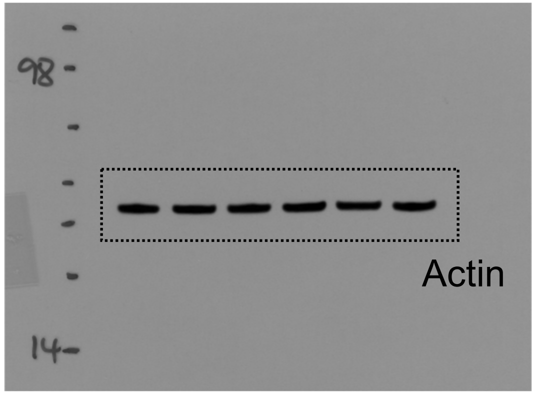


Supplementary Fig. 11 continued

Supplementary Fig. 5B

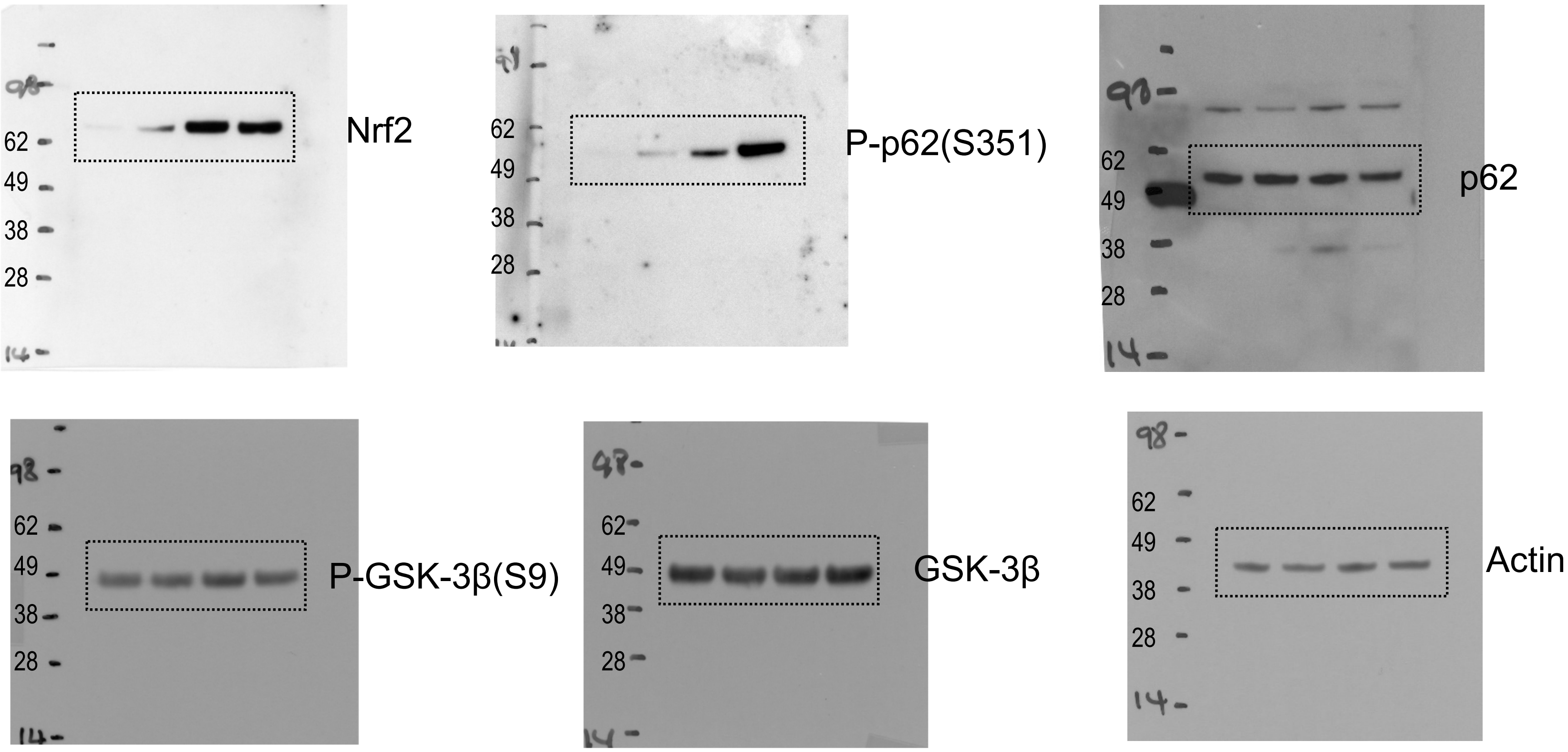


Supplementary Fig. 11 continued

Supplementary Fig. 6A

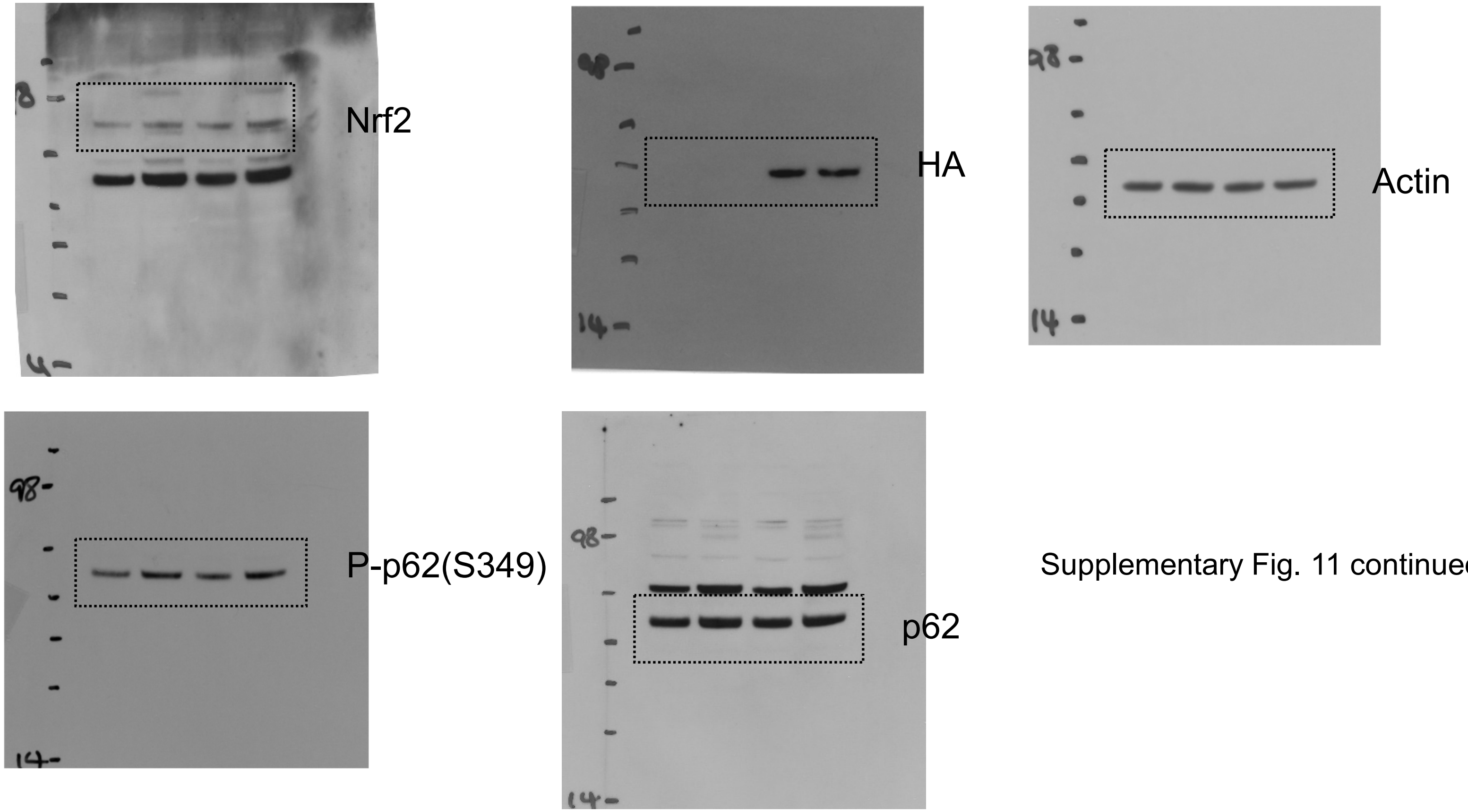


Supplementary Fig. 11 continued

Supplementary Fig. 6B

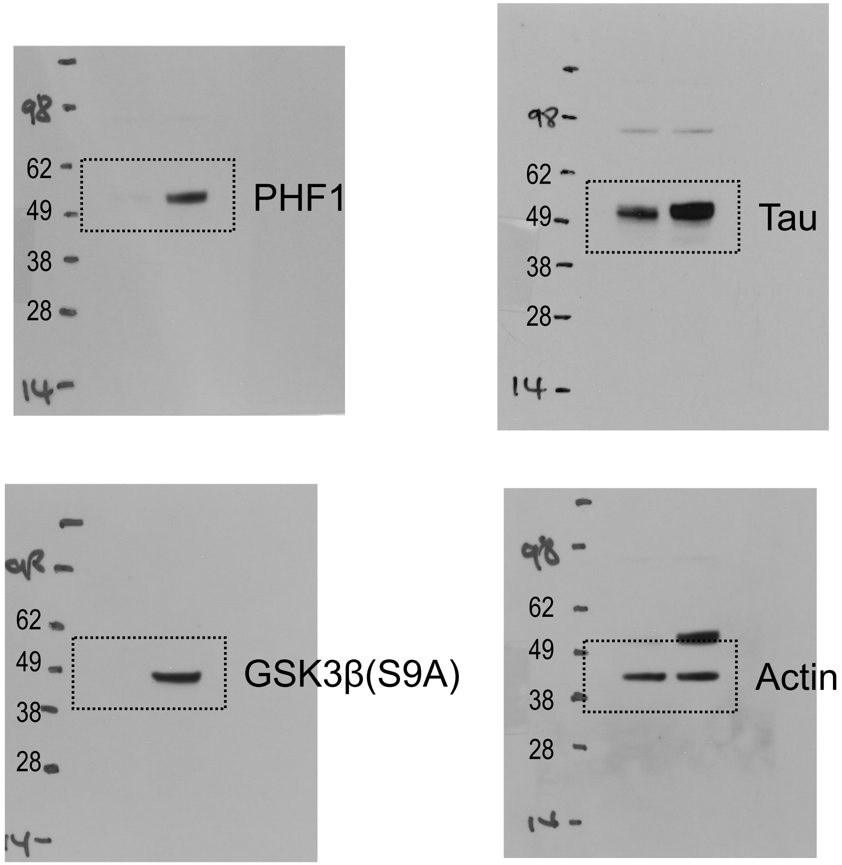


Supplementary Fig. 11 continued

Supplementary Fig. 7

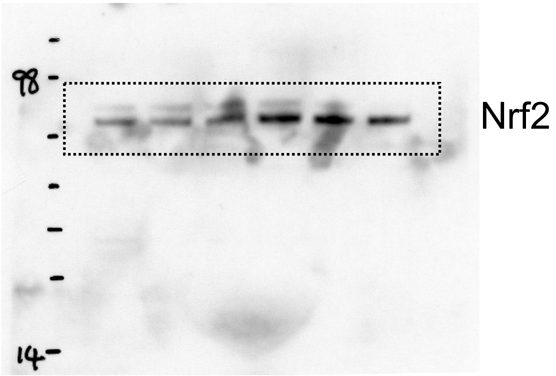


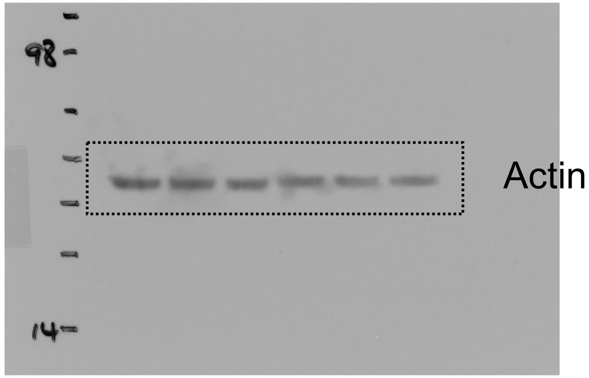


Supplementary Fig. 11 continued

Supplementary Fig. 8

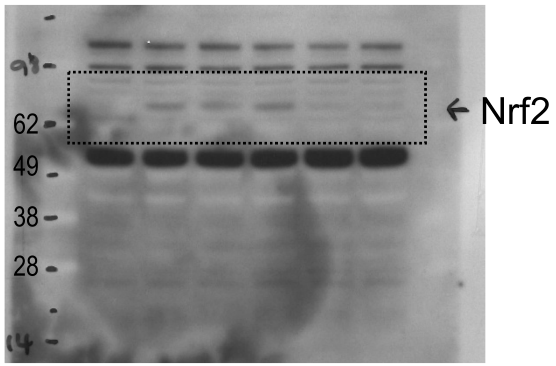


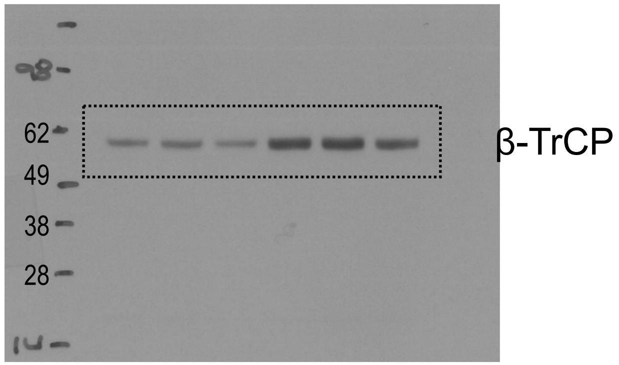


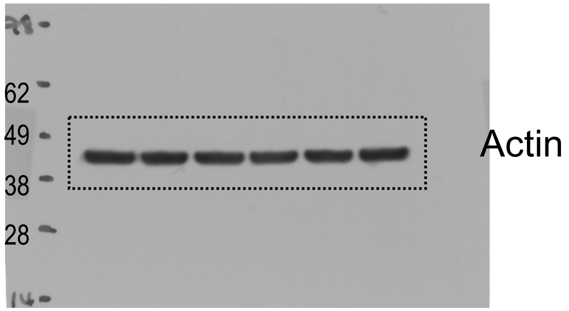


Supplementary Fig. 11 continued

Supplementary Fig. 9

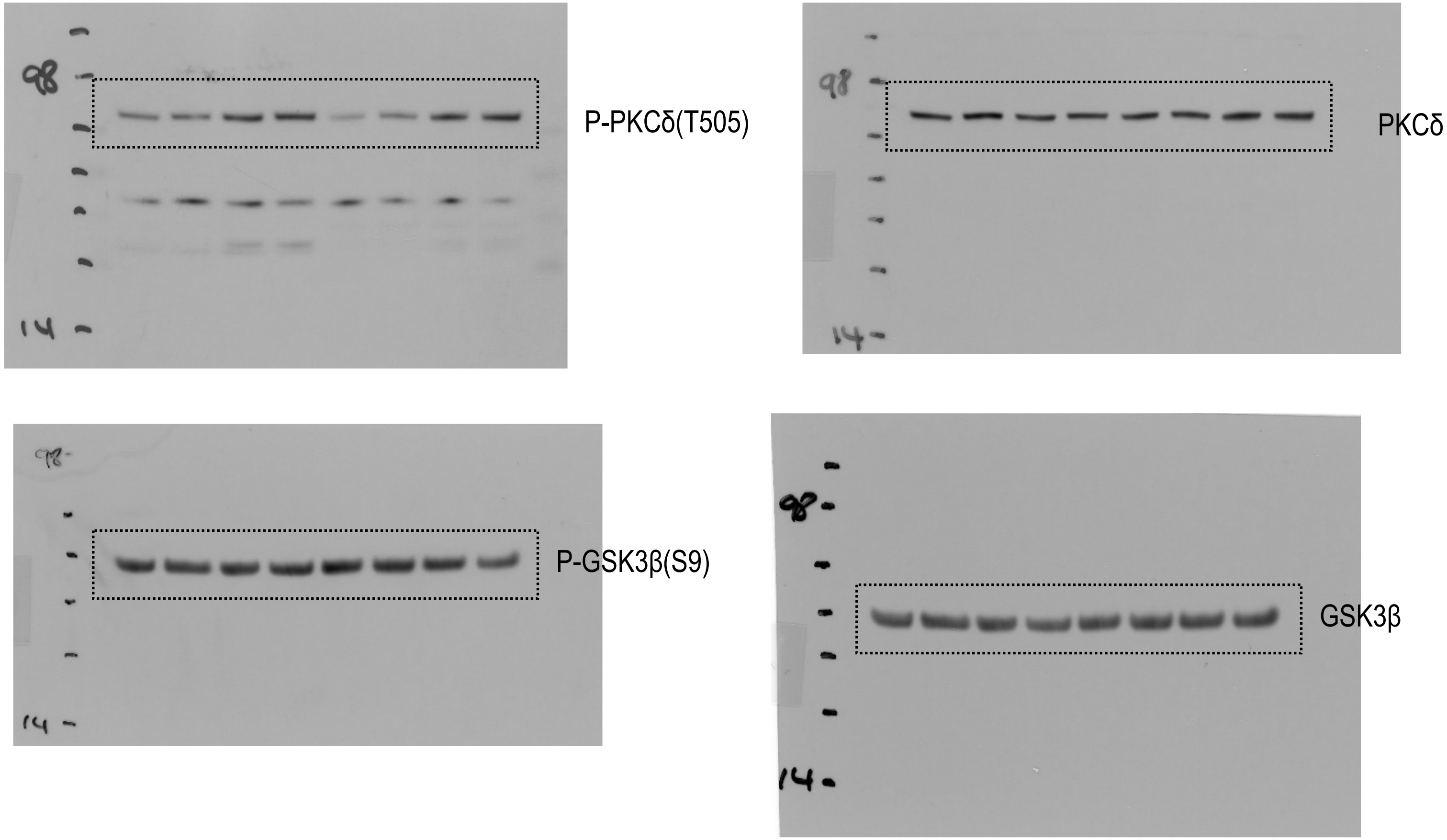


Supplementary Fig. 11 continued

Supplementary Fig. 10

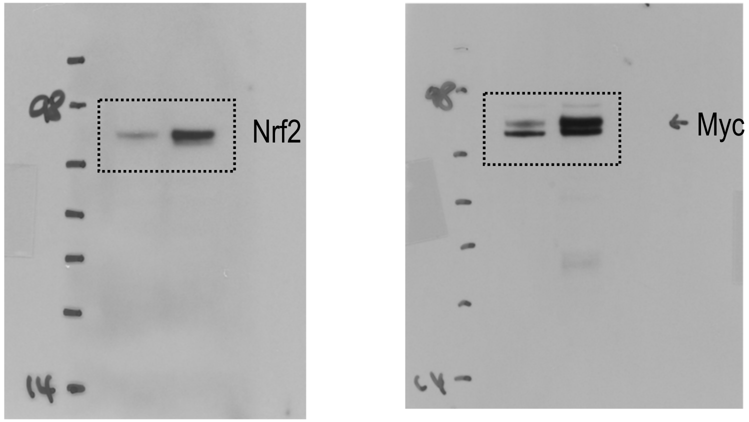


Supplementary Fig. 11 continued
